# Short- and long-term dietary supplementation as well as withdrawal of the enteric methane inhibitor 3-nitrooxypropanol reveal distinct effects on the rumen microbial community

**DOI:** 10.1186/s40104-025-01291-w

**Published:** 2025-12-01

**Authors:** Youyoung Choi, Mi Zhou, Atmir Romero-Pérez, Karen A. Beauchemin, Stephane Duval, Maik Kindermann, Le Luo Guan

**Affiliations:** 1 Faculty of Land and Food Systems, The University of British Columbia, Vancouver, BC V6T 1Z4 Canada; 2 Department of Agricultural, Food and Nutritional Science, University of Alberta, Edmonton, AB T6G 2P5 Canada; 3 Department of Animal Nutrition and Biochemistry, Faculty of Veterinary Medicine and Animal Science, National Autonomous University of Mexico, Mexico City, 04510 Mexico; 4 Agriculture and Agri-Food Canada, Lethbridge Research and Development Centre, Lethbridge, AB T1J 4B1 Canada; 5 DSM Nutritional Products, Animal Nutrition and Health, Wurmisweg 576, Kaiseraugst, 4303 Switzerland

**Keywords:** Beef cattle, Methane mitigation, Microbial interactions, Rumen microbiota, 3-Nitrooxypropanol

## Abstract

**Background:**

The enteric methane inhibitor 3-nitrooxypropanol (3-NOP) inhibits the key enzyme in ruminal methanogenesis, but whether short-term (ST) and long-term (LT) dietary supplementation has similar effects on rumen microbiota in beef cattle and how microbes change after 3-NOP withdrawal have not been studied. This study investigated changes in rumen bacteria, archaea, and protozoa after ST and LT dietary supplementation and removal of 3-NOP using metataxonomic analysis.

**Results:**

A total of 143 rumen samples were collected from two beef cattle studies with 3-NOP supplementation. The ST study (95 samples) used eight ruminally cannulated beef cattle in a 4 × 4 Latin square design with four 28-d of 3-NOP treatments [mg/kg of dry matter (DM)]: control: 0, low: 53, med: 161, and high: 345. The LT study (48 samples) was a completely randomized design with two 3-NOP treatments [control: 0, and high: 280 mg/kg of DM) fed for 112-d followed by a 16-d withdrawal (without 3-NOP). Bacterial and archaeal communities were significantly affected by 3-NOP supplementation but limited effects on protozoal communities were observed. Under ST supplementation, the relative abundances of *Prevotella*, *Methanobrevibacter* *(Mbb.) ruminantium*, *Methanosphaera* sp. ISO3-F5, and *Entodinium* were increased (*Q* < 0.05), whereas those of *Mbb*. *gottschalkii* and *Epidinium* were decreased (*Q* < 0.05) with 3-NOP supplementation. In LT study, relative abundances of *Mbb. ruminantium*, and *Methanosphaera* sp. Group5 were increased (*Q* < 0.05), while those of *Saccharofermentans* and *Mbb. gottschalkii* were decreased (*Q* < 0.05) with 3-NOP supplementation. Comparison between 3-NOP supplementation and the withdrawal revealed increased relative abundances of *Clostridia* UCG-014 and *Oscillospiraceae* NK4A214 group and decreased those of *Eubacterium nodatum* group and *Methanosphaera* sp. Group5 (*P* < 0.05) after 3-NOP withdrawal. Further comparison of rumen microbiota between control and 3-NOP withdrawal showed significantly higher (*P* = 0.029) relative abundances of *Eggerthellaceae* DNF00809, p-1088-a5 gut group, and *Family XII* UCG-001 in control group while no significant differences were detected for archaea and protozoa. Microbial network analysis revealed that microbial interactions differed by both 3-NOP dose and durations.

**Conclusions:**

Both ST and LT supplementation affected overall rumen microbial profile, with individual microbial groups responded to 3-NOP supplementation differently. After 3-NOP withdrawal, not all microbes showed recovery, indicating that the 3-NOP driven shifts were only partially reversible. These findings provide an understanding of the effects of 3-NOP on rumen microbial communities and their adaptability to methane mitigation strategies.

**Supplementary Information:**

The online version contains supplementary material available at 10.1186/s40104-025-01291-w.

## Background

Methane (CH_4_) is a greenhouse gas (GHG) that contributes significantly to global warming. Enteric CH_4_ emissions from ruminants account for 5% of global GHG emissions and 17% of GHG emissions related to the global food system [1]. Methane from livestock contributes 30% of global anthropogenic CH_4_ emissions, and from livestock CH_4_ emissions, of which 88% comes from enteric fermentation [2]. Global animal product demand per capita is estimated to increase by 20% by 2050 compared to 2020 levels [3], and consequently CH_4_ emissions from ruminant livestock are predicted to rise substantially [4]. In Canada, it is estimated that enteric fermentation from beef cattle contributes 22 million tonnes of carbon dioxide equivalent, accounting for 39% of total agricultural GHG emissions [5]. While beef cattle are significant CH_4_ emitters, they provide high-quality proteins for human’s consumption. The recent Intergovernmental Panel on Climate Change (IPCC) Sixth Assessment Report (AR6, 2022) highlighted that mitigating CH_4_ emissions could reduce the global warming effect over a relatively short timescale due to the short atmospheric lifetime of CH_4_ [6, 7]. Therefore, targeting enteric CH_4_ emissions from beef cattle is considered an effective climate mitigation strategy.

Of the tested and validated enteric CH_4_ mitigation strategies, the CH_4_ inhibitor 3-nitrooxypropanol (3-NOP) has shown considerable potential in reducing enteric CH_4_ production from ruminants and it has been approved as a feed additive by many countries [8]. The mode of action of 3-NOP involves in inhibiting methanogenesis by deactivating methyl-coenzyme M reductase (MCR), the final enzyme in CH_4_ formation by rumen methanogenic archaea [9]. Previous studies have demonstrated the effectiveness of 3-NOP in reducing enteric CH_4_ emissions in beef cattle [10–14] and dairy cows [15–19] and meta-analyses report mean reductions in beef cattle enteric CH_4_ emissions by 17.5% [20]. However, the extent of CH_4_ reduction varied depending on factors such as animal type, diet composition, and 3-NOP dosage [21, 22]. Previously, both short-term (ST, 28-d; [13]) and long-term (LT, 112-d; [14]) dietary 3-NOP supplementation showed decreased enteric CH_4_ emissions and they were associated with altered rumen fermentation patterns characterized by enhanced propionate and butyrate proportions and decreased acetate proportions in growing beef cattle. As the rumen microbiota is mainly responsible for fermentation and methanogenesis, effects of 3-NOP supplementation on microbial abundance were also evaluated in both studies. In the ST study, different doses of 3-NOP supplementation did not affect the abundances of total bacteria, total methanogens and total protozoa [13] compared with the control; whereas in the LT study, supplementation 3-NOP increased that of the total protozoa and decreased that of total methanogen but did not affect abundances of total bacteria compared to the control [14], suggesting the rumen microbiota responded differently to these management approaches. Recently, metagenomic analysis showed that 3-NOP affected on rumen microbial composition and functions related to methanogenesis, particularly hydrogen (H_2_) metabolism in dairy cattle [23], but changes in rumen bacterial, archaeal, and protozoal communities after ST and LT dietary supplementation and removal of 3-NOP have not been investigated.

In the previous LT study [14], CH_4_ emissions, total microbial abundance, and individual volatile fatty acid (VFA) proportions returned to normal levels during the recovery period when 3-NOP was removed from the diet. While recovery of CH_4_ emissions following 3-NOP withdrawal has been reported and previous studies evaluated only total microbial abundances, the detailed microbial community composition and structure changes during recovery period have not been investigated. Understanding these compositional changes could reveal whether the altered microbial communities have beneficial or detrimental effects on the rumen fermentation beyond methanogenesis. We hypothesized that ST and LT 3-NOP supplementation would have different effects on rumen microbial ecology, including distinct changes in microbial communities and their recovery patterns. The objective of the present study was to compare the effects of ST and LT 3-NOP supplementation on rumen microbial communities (bacteria, archaea, and protozoa) and to assess the effects of removing 3-NOP from the diet on recovery patterns to better understand the mechanisms of 3-NOP-mediated CH_4_ reduction. Understanding changes in microbial compositions (bacteria, archaea, and protozoa) is crucial for optimizing 3-NOP application in beef cattle.

## Methods

### Animal experiment and sampling

This study was a companion study to the beef cattle studies reported by Romero-Pérez et al. [13, 14] conducted at the Agriculture and Agri-Food Canada Research Centre in Lethbridge, Alberta, Canada. The experimental protocols received approval from University of Alberta Animal Care and Use Committee for Livestock (Protocol No. short-term: AUP#1132; long-term: AUP#1234). The experiments were conducted following the guidelines of the Canadian Council of Animal Care (2011, Ottawa, ON, Canada).

Rumen samples were collected in both studies [13, 14], and detailed information of feed analysis was shown in Tables S1 and S2. Both studies used similar basal diets consisting of 40% concentrate and 60% barley silage (dry matter (DM) basis). For the ST study, 8 ruminally-cannulated Angus heifers (549 ± 64.3 kg [mean BW ± SD]) were used in a 4 × 4 Latin square design with four 28-d periods. Dietary treatments examined 3-NOP dietary inclusion at four concentrations (mg/kg of DM: control, 0; low, 53; med, 161; and high, 345). Rumen digesta samples were collected on d 14 of each period at 0, 6, and 12 h after feeding (total of 95 samples), and enteric CH_4_ was measured for individual animals housed in metabolic chambers for three consecutive days starting on d 18 of each period. The LT study used 8 rumen-cannulated Angus heifers (637 ± 16.2 kg [mean BW ± SD]) in a completely randomized design with two 3-NOP concentrations [control, 0 and high, 280 mg/kg of DM]. The experiment consisted of 146 d, including an 18-d covariate period without 3-NOP, four 28-d periods with 3-NOP, and a 16-d recovery period without 3-NOP. On d 12 of the covariate period, d 22 of each treatment period, and d 8 of the recovery period, rumen digesta samples were collected at 0, 3, and 6 h after feeding for VFA analysis with the 3 h samples used for microbial composition analysis (total of 48 samples). During the covariate period and at the end of each period and the end of the recovery period, CH_4_ was measured for three consecutive days using metabolic chambers. Methane emissions have been reported previously [13, 14] and were extracted for analysis in this study. Power analyses based on CH_4_ production data using G*Power 3 [24] showed that both the ST and LT studies (*n* = 8) achieved > 0.99 power at α = 0.05, confirming that the sample sizes were sufficient to detect meaningful treatment effects.

### Rumen microbial profiling using amplicon sequencing

Total DNA was extracted from rumen digesta samples (ST: 95 and LT: 48) using the repeated bead-beating plus column method and purified using QIAamp Fast DNA Stool Mini kit, with DNA concentration and quality measured using NanoDrop and 1% agarose gel, respectively. All of the diluted DNA samples (50 ng/μL) were sent to Génome Québec Innovation Centre (McGill University, Quebec, Canada) to amplify bacteria/archaea partial 16S rRNA genes respectively using primer pairs Bac9F/Bac515R: 5′-GAGTTTGATCMTGGCTCAG//CCGCGGCKGCTGGCAC-3′, Arc915aF/Arc1386R: 5′-AGGAATTGGCGGGGGAGCAC/GCGGTGTGTGCAAGGAGC-3′, and protozoa partial 18S rRNA gene with RP841F/Reg1302R: 5′-GACTAGGGATTGGARTGG/AATTGCAAAGATCTATCCC-3′ (adopted from Rumen Consensus Program, Ag/Research, New Zealand [25]). All of the amplicons were then subjected to pyrosequencing analysis using the 454 Titanium FLX (Roche).

### Analysis of rumen microbiota shifts in response to 3-NOP supplementation and withdrawal

The obtained sequence reads were processed using QIIME2 (v.2024.5, [26]). The 454 sequence files (fna) and quality files (qual) were converted to fastq format, imported into QIIME2 (v.2024.5), then cutadapt [27] was used to remove barcodes and primer sequences for each target group (bacteria, archaea, and protozoa), respectively. Sequence denoising and clustering were performed using DADA2 [28] with ‘qiime dada2 denoise-pyro’ command. The merged representative sequences were then aligned against two reference databases: the Silva (v.138.1, [29]) for bacteria and the RIM-DB [30] for archaea. To classify the taxonomic identity of protozoal amplicon sequence variants (ASVs), we used BLASTn searches against the NCBI nucleotide database (NCBI-nt, accessed March 18, 2025), excluding sequences derived from uncultured organisms and environmental samples. The taxonomic identity of each protozoal ASV was established by selecting the top-scoring alignment result from the NCBI-nt database BLASTn analysis. The relative abundance of each taxon was determined as its proportion within the total taxa counts in the amplicon sequence variant (ASV) table for each sample. We additionally estimated the absolute abundance of each microbial taxon by multiplying relative abundance values by total bacteria, methanogens, and protozoa gene copy numbers [31]. Alpha diversity metrics, including Chao1 and Shannon indices, and Good’s coverage were analyzed at ASV level using QIIME2 (v.2024.5). Beta diversity was assessed at the ASV levels using both weighted and unweighted UniFrac distance metrics. Permutational multivariate analysis of variance (PERMANOVA) was used to test for significant differences among 3-NOP levels in the ST and LT studies using the adonis function of R package vegan (v. 2.6-6.1) and results were visualized using ggplot2 (v. 3.5.1).

### Combined analysis of metataxonomic data from short-term and long-term studies

A combined analysis approach was employed using MMUPHin (v.1.18.1, Meta-analysis methods with uniform pipeline for heterogeneity in microbiome studies [32]) to investigate 3-NOP effects across both studies while controlling for batch effects. Taxa with ≥ 0.05% abundance in at least 50% of animals within each group were retained for analysis. The "adjust_batch" function corrected for study-specific batch effects.

### Predicted functional analysis using CowPI and PICRUSt2-SC

To investigate the functional potential of rumen microbes, their metabolic profiles were predicted using CowPI [33] based on KEGG level 3 pathways, considering major functional features (≥ 0.05% relative abundance in more than 50% of animals). Additionally, PICRUSt2-SC (v2.6.1, released March 04, 2025) [34] was used to predict potential functions of bacteria and archaea. Five functional databases, including KEGG Orthology (KO), Enzyme Commission (EC) numbers, MetaCyc pathways, KEGG modules, and KEGG pathways, were employed using the same filtering criteria. Following the developers’ recommendations, bacterial and archaeal sequences were placed into their respective reference phylogenies. The results from both analyses were then combined for downstream analysis.

### Construction and topological characterization of co-occurrence networks in the rumen microbiota

Microbial co-occurrence networks were constructed using the genus-level relative abundance matrix based on Spearman’s correlation (|*r*| > 0.7, *P* < 0.05), calculated using the Hmisc (v.5.2-2) package in R. Topological indices including modularity (modularity > 0.4, [35]), average degree [36], and clustering coefficient [37] were calculated using the igraph (v.2.1.4) and tidygraph (v.1.3.1) packages to assess network complexity, as described in Pan et al. [38]. Network visualizations were performed using ggraph (v.2.2.1). Furthermore, within-module connectivity (Zi) and among-module connectivity (Pi) were computed to determine the topological roles of microbial taxa. Based on these values, taxa were classified into four roles: network hubs (Zi > 2.5, Pi > 0.62), module hubs (Zi > 2.5, Pi < 0.62), connectors (Zi < 2.5, Pi > 0.62), and peripherals (Zi < 2.5, Pi < 0.62) [39]. Further, module eigengenes were correlated with CH_4_ emission data [g/d and g/kg of DM intake (DMI)] from Romero-Pérez et al. [13, 14] to explore potential associations. The co-occurrence network analysis was only conducted for ST and LT studies and not for the recovery groups due to insufficient sample size.

### Relationships between rumen microbial taxa, fermentation parameters, and CH_4_ emission

Spearman’s correlation analysis between the selected microbial taxa (bacteria, archaea, and protozoa) and phenotype parameters was conducted using the PROC CORR procedure in SAS and visualized using R package pheatmap (v. 1.0.12), with rumen fermentation parameters [pH, total VFA, molar proportion of VFAs, and acetate to propionate ratio (AP)] and CH_4_ emission data (g/d and g/kg of DMI) from Romero-Pérez et al. [13, 14]. Correlation coefficients were considered significant at |*r*| ≥ 0.30 and *P* < 0.05.

### Statistical analysis

Statistical analyses were performed using SAS (v.9.4, SAS Institute Inc., Cary, NC, USA) and R software (v.4.2.0). Alpha diversity measurements were compared among treatment groups using different statistical methods for each study (Kruskal-Wallis test followed by Dunn’s test for the ST study; Wilcoxon rank-sum test for the LT study), which were performed using R software stats (v.4.2.0) and FSA (v.0.9.5, Fisheries Stock Assessment). For both studies, differential abundance analysis was conducted on microbial taxa (relative abundance ≥ 0.05% in more than 50% of animals) to avoid spurious results and improve the robustness of comparisons. Differential abundance analyses were conducted using MaAsLin2 (v.1.12.0, Microbiome Multivariable Associations with Linear Models, [40]), which applied linear models (LM) for bacterial taxa and compound Poisson linear models (CPLM) for archaeal and protozoal taxa. This was based on their distribution characteristics, particularly the degree of zero inflation and variance to mean ratio. In the ST study, comparisons were made between the control and 3-NOP supplementation groups, with period and sampling time points included as fixed effects. For the LT study, 3-NOP treatment was used as a fixed effect to compare control and 3-NOP supplementation, while the Wilcoxon rank-sum test (R package stats) with averaged data was used to compare 3-NOP supplementation and recovery periods. Significant differences in taxa were determined at *Q* < 0.05 in MaAsLin2 [40], where the coefficients represent the effect size of a variable on the response and *Q* value indicates the minimum false discovery rate (FDR). Statistical significance was determined at *Q* < 0.05 and tendencies were considered at 0.05 ≤ *Q* < 0.1.

## Results

### Effects of short- and long-term 3-NOP supplementation of beef cattle diets on microbial communities

**Fig. 1:**
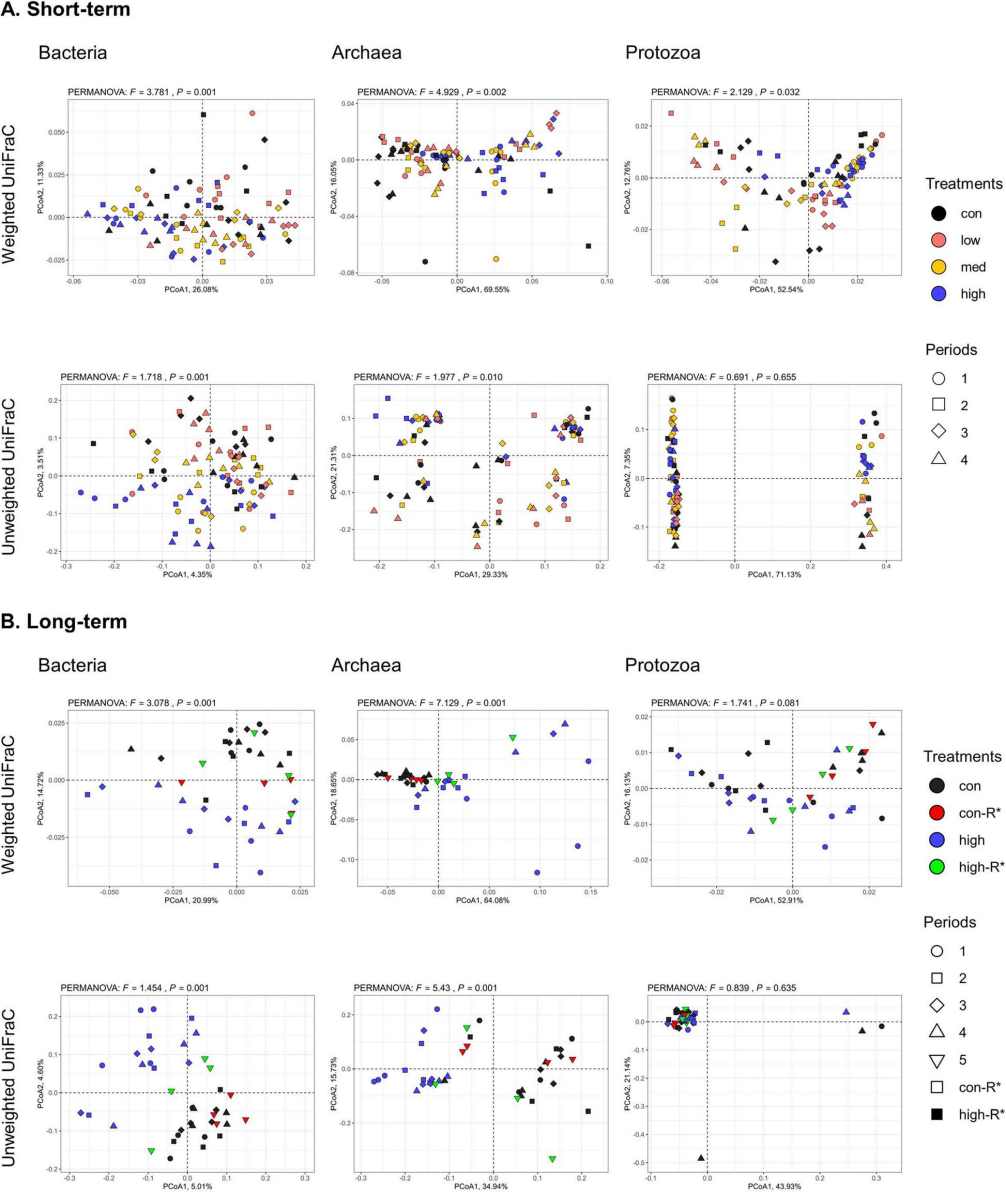
Comparison of beta diversities of bacterial, archaeal, and protozoal communities after 3-nitrooxypropanol (3-NOP) supplementation in beef cattle for **A** short- and **B** long-term periods. 3-NOP dose in the short-term study were: control: 0 (black), low: 53 (red), med: 161 (yellow), high: 345 (blue) mg/kg of DM. 3-NOP dose in the long-term study were: control: 0 (black), control-R*: 0 (deep red), high: 280 (blue), high-R*: 0 (green) mg/kg of DM. Legend: each symbol represents different treatments and periods (1: d 1 to 28; 2: d 29 to 56; 3: d 57 to 84; 4: d 85 to 112). PCoA1 and PCoA2 axes are shown with the percentages of variation they explain. control: control; DM: dry matter; R*: recovery period

After removing sequencing effects and performing quality filtration (Q-score > 25) of the data, the average sequence counts (mean ± standard error) were: bacterial (ST: 6,908 ± 245, LT: 6,883 ± 460), archaeal (ST: 1,481 ± 35, LT: 1,043 ± 102), and protozoal (ST: 1,591 ± 48, LT: 845 ± 112). Good's coverage indices were 99.9% for three microbial groups in all rumen samples, indicating that the sequencing depth was sufficient to capture the diversity of the rumen microbial community. Consistently, rarefaction curves showed that bacterial, archaeal, and protozoal communities plateaued at the obtained sequencing depths (Fig. S1). As shown in Table 1, 3-NOP supplementation in the ST study significantly decreased archaeal richness (*P* = 0.018) and increased protozoal evenness (*P* = 0.029) at the high dose compared to the control. In the LT study, archaeal richness tended to be lower (*P* = 0.063) in the high group compared to control group, but showed no differences after recovery (high-R*) between control and high-R* and between high and high-R* groups (Table 2). Bacterial evenness tended to be higher in the high-R* group than in the high group (*P* = 0.057), with no significant differences between control and high or between control and high-R* groups. Beta diversity analysis based on weighted and unweighted UniFrac metrics showed significant overall differences across all treatment groups in the ST study. Both bacteria and archaea showed significant differences in unweighted (*P* = 0.001 and *P* = 0.002) and weighted UniFrac metrics (*P* = 0.001 and *P* = 0.010, respectively), while protozoa showed significant differences only in unweighted UniFrac (*P* = 0.032) but not in weighted UniFrac (*P* = 0.655) (Fig. 1A). Similarly, in the LT study, bacteria and archaea showed significant differences in both unweighted and weighted UniFrac (*P* = 0.001 for all), whereas protozoa showed a tendency between control and high in unweighted UniFrac (*P* = 0.081) but not in weighted UniFrac (*P* = 0.635) (Fig. 1B). Detailed results on the pairwise comparison tests are presented in Tables S3 and S4. Further, we performed combined analysis across both studies, after correcting for study-specific batch effects, which revealed consistent 3-NOP-associated shifts in microbial composition. Bacterial and archaeal communities showed significant differences between control and 3-NOP groups (bacteria: *P* = 0.001; archaea: *P* = 0.002), whereas protozoal communities had no significant difference (Fig. S2).

**Table 1:** Alpha diversity measurements of the rumen microbiota after short-term 3-nitrooxypropanol (3-NOP) supplementation in beef cattle

Measurement	Con	3-NOP^1^			SEM	*P* value
		Low	Med	High		
Chao1 index						
Bacteria	416	447	412	408	11.7	N.S
Archaea	12.5^a^	12.3^a^	12.6^a^	9.88^b^	0.35	0.018
Protozoa	15.8	17.7	15.9	15.9	0.35	N.S
Shannon index						
Bacteria	0.904	0.917	0.916	0.908	0.00	N.S
Archaea	0.769	0.699	0.770	0.718	0.01	N.S
Protozoa	0.820^b^	0.817^b^	0.830^ab^	0.860^a^	0.01	0.029

*3-NOP* 3-Nitrooxypropanol, *SEM* Standard error of the mean, *DM* Dry matter, *Con* Control, *N.S* Not significant

^1^3-NOP dose: Con: 0, Low: 53, Med: 161, High: 345 mg/kg of DM

^a,b^Values with different letters differ significantly (*P* < 0.05)

**Table 2:** Alpha diversity measurements of the rumen microbiota after long-term 3-nitrooxypropanol (3-NOP) supplementation in beef cattle

Measurement	Treatments^1^			Con vs. High		Con vs. High-R*		High vs. High-R*	
	Con	High	High-R*	SEM	*P* value	SEM	*P* value	SEM	*P* value
Chao1 index									
Bacteria	538	570	612	20.8	N.S	37.5	N.S	35.2	N.S
Archaea	12.0	10.2	10.5	0.48	0.063	0.94	N.S	0.89	N.S
Protozoa	10.3	10.8	12.3	0.54	N.S	0.79	N.S	0.73	N.S
Shannon index									
Bacteria	0.910	0.900	0.917	0.00	N.S	0.00	N.S	0.00	0.057
Archaea	0.750	0.638	0.680	0.02	N.S	0.03	N.S	0.03	N.S
Protozoa	0.800	0.810	0.873	0.02	N.S	0.02	N.S	0.02	N.S

*3-NOP* 3-Nitrooxypropanol, *SEM* Standard error of the mean, R* Recovery period, *Con* Control, *N.S* Not significant

^1^3-NOP dose level information long-term: Con: 0, High: 280, High-R*: 0 mg/kg of DM

### Effects of short-term 3-NOP supplementation of beef cattle diets on rumen microbial composition

**Fig. 2:**
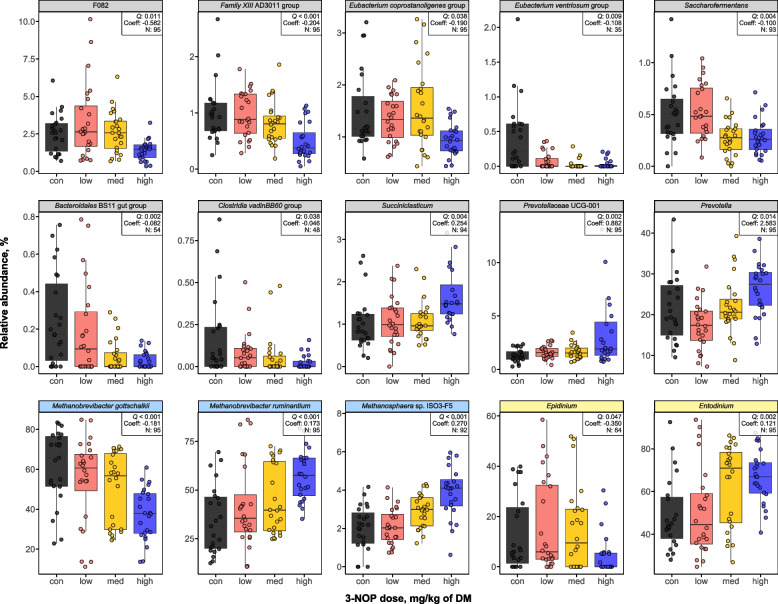
Comparison of rumen microbial taxa (bacteria, archaea, and protozoa) identified after short-term 3-nitrooxypropanol (3-NOP) supplementation in beef cattle. Taxa were selected based on significant treatment effects (*Q* < 0.05) from MaAsLin2 analysis. Genera with a Benjamini-Hochberg false discovery rate-adjusted* Q* < 0.05 were considered statistically significant for bacteria, archaea, and protozoa. The relative abundance of major phyla and genera (≥ 0.05% in more than 50% of animals) was analyzed for all individuals. The gray, skyblue, and yellow strips represent bacteria, archaea, and protozoa, respectively. 3-NOP doses used in the short-term study were: control: 0 (black), low: 53 (red), med: 161 (yellow), high: 345 (blue) mg/kg of DM. control: control; UCG: uncultured genus-level; DM: dry matter

A total of 84 bacterial genera were observed in the ST study with 60 genera shared between animals regardless of 3-NOP dose with exclusive genera found for control (*Eubacterium ventriosum* group), low (*Lachnobacterium* and *Pyramidobacter*) and medium (p-1088-a5 gut group, UG Selenomonadaceae, and *Oscillospiraceae* UCG-005) doses but none for the high dose (Fig. S3a). Among bacteria, 16 genera were significantly (*Q* < 0.05) different from control after 3-NOP supplementation with 11 genera decreasing (including *Eubacterium ventriosum* group, *Bacteroidales* BS11 gut group, *Clostridia* vadinBB60 group, and *Saccharofermentans*) and 5 genera increasing (including *Prevotella*, *Succiniclasticum*, *Prevotellaceae* UCG-001) (Fig. 2). In the estimated absolute abundance analysis, fewer bacterial taxa remained significant, including decreases in *Oscillospiraceae* V9D2013 group and increases in *Clostridium *sensu stricto 1 and *Succinivibrio* (Table S5). Four archaeal species were observed in total with 3 species in common for all dose levels and one archaeal species (*Methanomassiliicoccaceae* Group12 sp. ISO4-H5) was found exclusively in the high group (Fig. S3b). Based on relative abundance, three archaeal species differed significantly (*Q* < 0.05) from the control after 3-NOP supplementation with *Methanobrevibacter (Mbb.) gottschalkii* decreased and *Mbb*. *ruminantium* and *Methanosphaera* sp. ISO3-F5 increased (Fig. 2), whereas based on estimated absolute abundance, only *Mbb*. *gottschalkii* showed a significant decreased (Table S5). Seven protozoal genera were observed with 5 genera (*Diploplastron*, *Entodinium*, *Isotricha*, *Ostracodinium*, and *Polyplastron*) in common for all dose levels and one genus (*Dasytricha*) found only in the 3-NOP supplementation groups (Fig. S3c). Two genera differed significantly (*Q* < 0.05) from control after 3-NOP supplementation where *Epidinium* decreased and *Entodinium* increased (Fig. 2), whereas the estimated absolute abundance of *Ostracodinium* increased and that of *Epidinium* decreased (Table S5).

The relative abundances of bacteria and protozoa at the genus level and archaea at species level are presented as pie charts for ST study (Fig. S4). *Prevotella*, *Christensenellaceae* R-7 group, and *Ruminococcus* were predominant in both control and 3-NOP supplemented groups, with *Prevotella* showing increased relative abundance in the high group (control: 21.6%, low: 17.5%, med: 22.2%, and high: 26.3%). For archaea, *Mbb*. *gottshalkii* was the most abundant species in the rumen of the control group, while in the high group, *Mbb*. *ruminantium* showed higher relative abundance than *Mbb*. *gottshalkii*. Notably, *Methanosphaera* sp. ISO3-F5 showed much higher relative abundance in the high group (control: 2.0% and high: 4.0%). Among protozoa, *Entodinium* was the dominant genus in the rumen of all groups (control: 49.2%, low: 50.5%, med: 62.8%, and high: 66.8%), and *Diploplastron* (control: 13.2%, low: 8.4%, med: 5.3%, and 7.9%) and *Epidinium* (control:13.2%, low: 16.0%, med: 15.0%, and high: 4.9%) as the second and third most abundant genera, respectively.

### Effects of long-term 3-NOP supplementation of beef cattle diets on rumen microbial composition

**Fig. 3:**
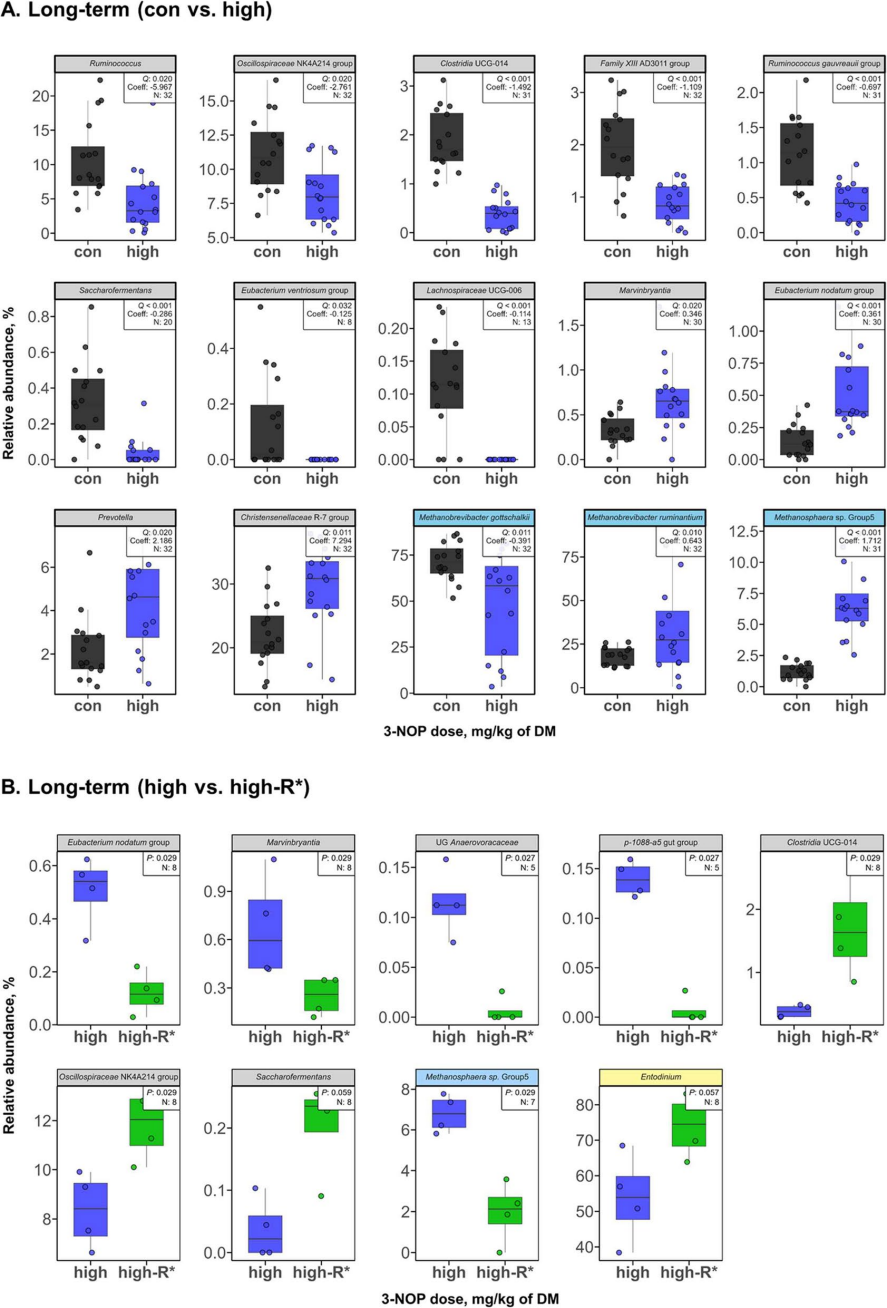
Comparison of rumen microbial taxa (bacteria, archaea, and protozoa) identified after long-term 3-nitrooxypropanol (3-NOP) supplementation in beef cattle. **A** Control vs. high (3-NOP) groups where taxa were selected based on MaAsLin2 analysis with Benjamini-Hochberg false discovery rate-adjusted *Q* < 0.05 considered statistically significant for bacteria and archaea. **B** The high vs. recovery period (high-R*) groups where taxa with *P* < 0.05 in Wilcoxon signed-rank test were considered statistically significant. The relative abundance of major phyla and genera (≥ 0.05% in more than 50% of animals) was analyzed for all individuals. The gray and skyblue strips represent bacteria and archaea, respectively. R* indicates the recovery period after discontinuing 3-NOP supplementation. 3-NOP doses used in the long-term study were: control: 0 (black), high: 280 (blue), high-R*: 0 (green) mg/kg of DM. UCG: uncultured genus-level; UG: unclassified genus-level; DM: dry matter; R*: recovery period

In the LT study, 62 bacterial genera were observed with 55 genera shared between control and high groups, with exclusive genera found in control (*Atopobiaceae*, *Treponema*, UCG *Paracaedibacteraceae*, *Lachnospiraceae* UCG-006, *Defluviitaleaceae* UCG-011, and *Saccharofermentans*) and high (*Enterohabdus*) groups (Fig. S3d). Sixteen bacterial genera were significantly (*Q* < 0.05) different from control after 3-NOP supplementation with 10 genera decreasing (including *Ruminococcus*, *Ruminococcus gauvreauii* group, and *Saccharofermentans*) and 6 genera increasing (including *Oscillospiraceae* UCG-005, *Eubacterium nodatum* group, and *Prevotella*) (Fig. 3A). The estimated absolute abundance of nine bacterial genera differed significantly (*Q* < 0.05) from the control, with three genera decreasing (*Defluviitaleaceae* UCG-011, *Lachnospiraceae* UCG-006, and *Saccharofermentans*) and six genera increasing (*Prevotella*, *Muribaculaceae*, *Eubacterium hallii* group, *Christensenellaceae* R7 group, UCG *Lachnospiraceae*, and *Rikenellaceae* RC9 gut group) (Table S6). Four archaeal species were observed with 3 species shared and *Mbb*. *boviskoreani* found only in the control group (Fig. S3e), while three species showed significant differences (*Q* < 0.05) from control after 3-NOP supplementation, where *Mbb*. *gottschalkii* decreased and *Mbb*. *ruminantium* and *Methanosphaera* sp. Group5 increased (Fig. 3A). Based on the estimated absolute abundance analysis, *Mbb*. *gottschalkii* and *Mbb*. *boviskoreani* decreased significantly (*Q* < 0.05) (Table S6). Six protozoal genera were observed and four were shared (*Dasyticha*, *Isotricha*, *Entodinium*, and *Ophryoscolex*) (Fig. S3f) with no significant differences observed between control and high groups, while *Diploplastron* and *Ostracodinium* were exclusively detected in the high groups; however, the absolute abundance of *Ostracodinium* increased significantly (*Q* < 0.05) (Table S6).

The relative abundances of bacteria and protozoa at the genus level and archaea at species level are presented as pie charts for LT study (Fig. S5). *Christensenellaceae* R-7 group, *Lachnospiraceae* NK3A20 group, and *Oscillospiraceae* NK4A214 group were predominant in both control and 3-NOP supplemented groups, with *Christensenellaceae* R-7 group showing increased relative abundance in the high group (control: 22.8% and high: 30.1%). Similar to the ST study, *Mbb*. *gottshalkii* was enriched in the control group, while *Mbb*. *ruminantium* was more abundant in the high group. *Methanosphaera* sp. Group5 showed much higher relative abundance in the high group (control: 1.21% and high: 6.71%). Among protozoa, *Entodinium* was the dominant genus in the rumen of both groups (control: 60.3% and high: 52.8%), and *Ophryoscolex* was present in large proportions with similar relative abundances.

### Effects of 3-NOP withdrawal after long-term supplementation on rumen microbial composition in beef cattle

Comparison between control and high-R* groups contained 68 bacterial genera with 63 genera shared between groups and exclusive genera found in control (*Eubacterium ventriosum* group, UG *Anaerovoracaceae*, *Eggerthellaceae* DNF00809, and p-1088-a5 gut group) and high-R* (*Succiniclasticum*) groups (Fig. S3g). Six archaeal species were observed, with five shared between the control and high-R* groups (Fig. S3h) and *Methanosphaera* sp. ISO3-F5 found exclusively in the control group. Among the protozoal genera, six were observed with five shared (*Dasytricha*, *Isotricha*, *Entodinium*, *Ophryoscolex*, and *Ostracodinium*), and *Diploplastron* was found exclusively in the high-R* group (Fig. S3i). In the control group, the relative abundance of *Eggerthellaceae* DNF00809, p-1088-a5 gut group, and *Family XII* UCG-001 were significantly higher (*P* = 0.029), whereas no significant differences were observed in archaea and protozoa between two groups. The estimated absolute abundance of *Eggerthellaceae* DNF00809 and p-1088-a5 gut group were significantly higher in the control group (*P* = 0.027) (Table S7). As shown in Fig. S6, *Christensenellaceae* R-7 group, *Lachnospiraceae* NK3A20 group, and *Oscillospiraceae* NK4A214 group remained the predominant bacterial genera in both control and high-R* groups, with *Christensenellaceae* R-7 group maintaining higher relative abundance in the high-R* group (control: 22.7% and high-R*: 29.3%). For archaea, *Mbb*. *gottshalkii* (control: 72.0% and high-R*: 77.0%) and *Mbb*. *ruminantium* (control: 17.8% and high-R*: 19.2%) dominated the archaeal composition in both groups with similar relative abundance. The protozoal relative abundance mainly consisted of *Entodinium* (control: 59.2% and high-R*: 74.0%) and *Ophryoscolex* (control: 33.8% and high-R*: 14.8%), with different proportions between groups.

The high and high-R* groups revealed 68 bacterial genera with 59 genera shared between groups and exclusive genera found in high (*Eggthellaceae* DNF00809, *Enterorhabdus*, UG *Anaerovoracaceae*, and p-1088-a5 gut group) and high-R* (*Defluviitaleaceae* UCG-011, *Lachnospiraceae* UCG-006, *Pirellula*, *Bacteroidales* UCG-001, and CAG-352) groups (Fig. S3j). Six bacterial genera were significantly (*P* < 0.05) different between the high 3-NOP and recovery groups with 4 genera decreased (including UG *Anaerovoracaceae*, *Eubacterium nodatum* group, p-1088-a5 gut group, and *Marvinbryantia*) and 2 genera increased (including *Clostridia* UCG-014 and *Oscillospiraceae* NK4A214 group) (Fig. 3B). Additionally, *Saccharofermentans* tentatively increased (*P* = 0.057) in the recovery group. Based on the estimated absolute abundance analysis, *Eubacterium ventriosum* group and *Lachnospiraceae* UCG-006 decreased, whereas increases were found for *Clostridium methylpentosum* group and UCG *Erysipelotrichaceae* (*P* < 0.05) (Table S8). Five archaeal species were observed with 4 species shared (Fig. S3k) and US *Mbb.* found only in the high-R* group, while *Methanosphaera* sp. Group5 significantly (*P* = 0.029) decreased (Fig. 3B) in the high-R* group. Six protozoal genera were observed and shared (*Daystricha*, *Isotricha*, *Diploplastron*, *Entodinium*, *Ophryoscolex*, and *Ostracodinium*) (Fig. S3l) with no significant differences between groups, but *Entodinium* had a tentative increase (*P* = 0.057) in the high-R* group. Regarding relative abundances in the high vs. high-R* comparison, the same top three bacterial genera remained dominant as observed in the previous comparisons, with *Christensenellaceae* R-7 group showing similar relative abundance in both high (30.1%) and high-R* (29.3%) groups (Fig. S7). For archaea, *Mbb*. *gottshalkii* (high: 48.3% and high-R*: 77.0%), *Mbb*. *ruminantium* (high: 34.6% and high-R*: 19.2%), and *Methanosphaera* sp. Group5 (high: 6.8% and high-R*: 2.0%) showed notable shifts in relative abundance after withdrawal of 3-NOP supplementation. Among protozoa, *Entodinium* was higher in the high-R* group (high: 53.7% and high-R*: 74.0%) while *Ophryoscolex* was lower (high: 32.3% and high-R*: 14.8%).

### Batch effect correction and cross-study investigation of 3-NOP effects on rumen microbial communities in beef cattle

Batch effect correction was applied to integrate the ST and LT datasets and reduce study-specific variation [32]. This approach enabled clearer identification of 3-NOP effects and improved the detection of consistent associations across the two experimental designs. The combined analysis after batch effect correction confirmed that 3-NOP affected rumen microbial taxa including bacteria, archaea, and protozoa as determined from relative abundance (Fig. S8). A total of 24 genera were affected by 3-NOP supplementation, including 18 that were significant (*Q* < 0.05) and 6 that showed a tendency (0.05 ≤ *Q* < 0.1). Among these were 20 bacterial genera, with 12 decreased (e.g., *Clostridia vadinBB60* group, *Lachnospiraceae* UCG-006, and *Bacteroidales* BS11 gut group) and 8 increased (e.g., *Prevotella*, *Succiniclasticum*, and *Anaerovibrio*). Three archaeal genera were also affected, with *Mbb*. *gottschalkii* decreased while *Mbb*. *ruminantium* and *Methanosphaera* sp. Group5 increased. In addition, one protozoal genus, *Epidinium*, tended to decrease (*Q* = 0.074).

### Effects of 3-NOP supplementation on predicted rumen microbial functions in beef cattle

**Fig. 4:**
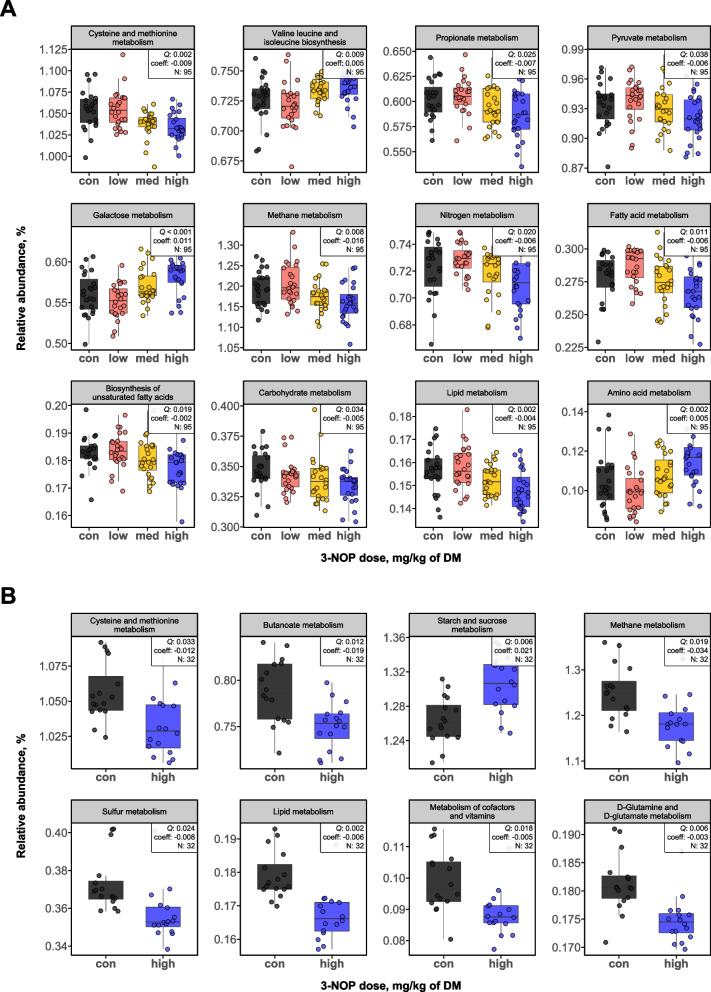
Differentially abundant predicted microbial functions after **A** short-term and **B** long-term after 3-nitrooxypropanol (3-NOP) supplementation in beef cattle. Functional features were identified using CowPI based on bacterial 16S rRNA gene sequences. 3-NOP doses used in the short-term study were: control: 0 (black), low: 53 (red), med: 161 (yellow), high: 345 (blue) mg/kg of DM. 3-NOP doses used in the long-term study were: control: 0 (black), high: 280 (blue) mg/kg of DM. con: control; DM: dry matter

Functional profiles of the rumen microbiota were predicted using CowPI [33], which incorporates a rumen-specific database to improve accuracy in pathway inference. When comparing the control group to the 3-NOP supplemented groups in the ST study, a total of 65 pathways were affected (34 increased and 31 decreased; Table S8). Specifically, the metabolism of CH_4_ (*Q* = 0.008), nitrogen (*Q* = 0.020), carbohydrates (*Q* = 0.034), lipid (*Q* = 0.002), and propionate (*Q* = 0.025) were decreased after 3-NOP supplementation. Conversely, metabolism of amino acid (*Q* = 0.009) and galactose (*Q* = 0.001) and valine, leucine and isoleucine biosynthesis (*Q* = 0.009) were increased (Fig. 4A). For the LT study, when comparing the control group to the high group, a total of 36 pathways were affected (9 increased and 27 decreased; Table S9). Among them, metabolism of CH_4_ (*Q* = 0.019), sulfur (*Q* = 0.024), lipid (*Q* = 0.002), butanoate (*Q* = 0.012), D-glutamine and D-glutamate (*Q* = 0.006), and cysteine and methionine (*Q* = 0.033) were decreased, whereas starch and sucrose metabolism (*Q* = 0.006) was increased after 3-NOP supplementation (Fig. 4B). When comparing the high group and high-R* group, the phosphatidylinositol signaling system was decreased (*P* = 0.030, log_2_ fold change = −0.047), while comparison between the control group and high-R* group showed that DNA replication proteins were decreased (*P* = 0.030, log_2_ fold change = −0.014). To complement CowPI, we additionally used PICRUSt2-SC (v.2.6.1), which has been updated with GTDB r214 genomes and improved functional annotations. This version improves prediction accuracy, particularly for archaeal functions, and provides broader coverage of microbial pathways. Results for the ST and LT studies are presented in Fig. S9 and Fig. S10.

### Altered microbial interactions in response to short- and long-term 3-NOP supplementation and their associations with methane emissions

**Fig. 5:**
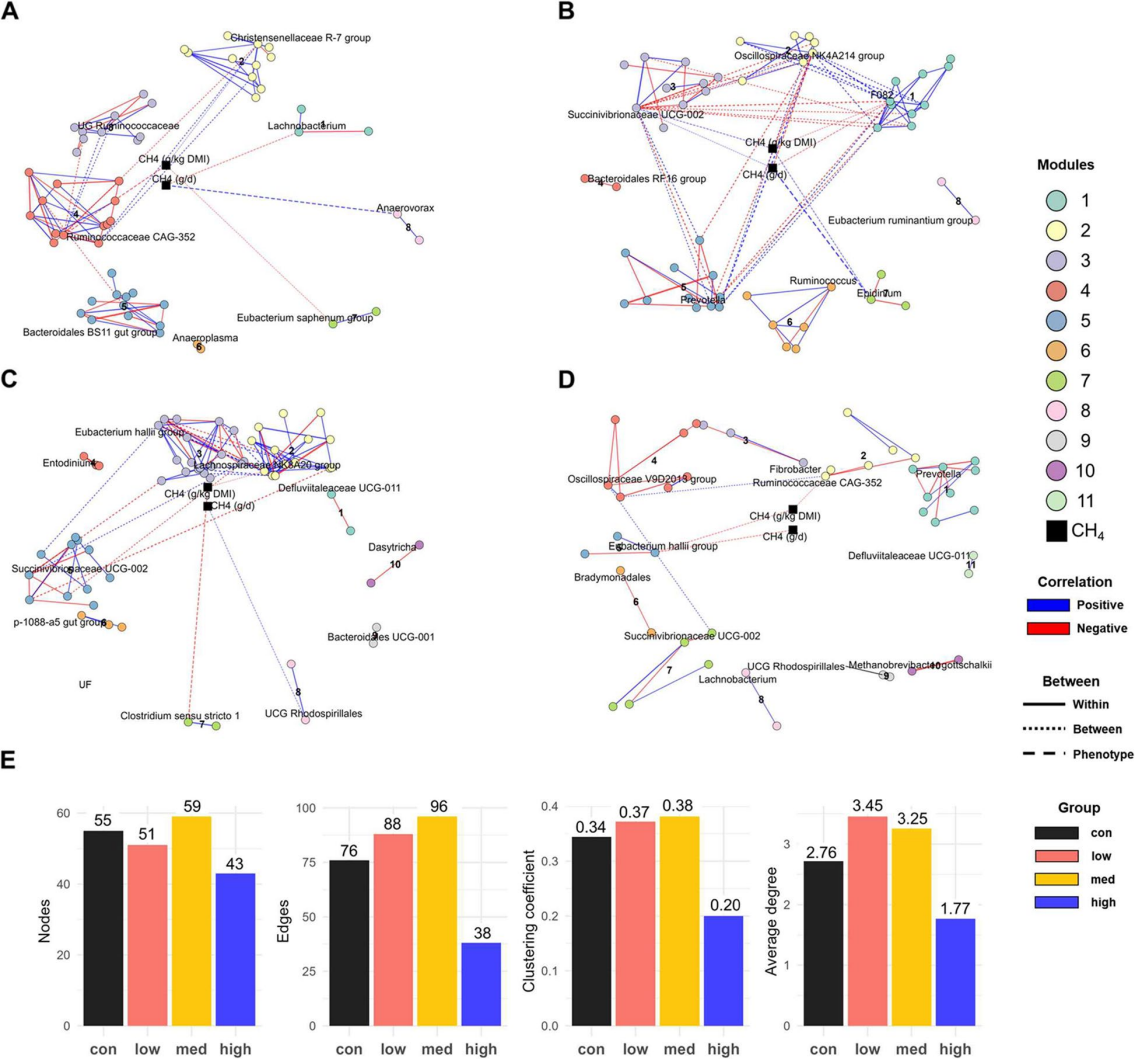
Co-occurrence networks of rumen microbial taxa after short-term 3-nitrooxypropanol (3-NOP) supplementation in beef cattle. **A**−**D** Microbial co-occurrence networks which were constructed based on Spearman’s correlation (|*r*| > 0.6, *P* < 0.05), and modular structures were identified. Node colors indicate module membership, with modules showing significant correlations to methane emission traits (CH_4_ g/d and CH_4_ g/kg DMI) connected to phenotype nodes (black). **E** The number of nodes, edges, clustering coefficient, and average degree for each treatment group. 3-NOP doses used in the short-term study were: control: 0 (black), low: 53 (red), med: 161 (yellow), high: 345 (blue) mg/kg of DM. CH_4_: methane; DMI: dry matter intake

To investigate microbial interactions in response to 3-NOP supplementation, co-occurrence networks were constructed for ST and LT studies based on Spearman’s correlation (|*r*| > 0.6, *P* < 0.05). In the ST study, all co-occurrence networks showed relatively high modularity (> 0.460), with the highest in the high 3-NOP group (0.784) and the lowest in the low group (0.460) (Table S10). As shown in Fig. 5A−D, co-occurrence networks comprising control (8), low (8), med (10), and high (11) modules were constructed, and the highest degree taxon in each module was annotated as a representative. Spearman correlation analysis was performed between each module and CH_4_ emission traits. In the control group, module 8 (represented by *Anaerovorax*) showed a positive correlation with CH_4_ emission (g/d; *r* = 0.716, *P* < 0.001), whereas module 4 (represented by *Ruminococcaceae* CAG-352) showed a negative correlation with CH_4_ emission (g/kg DMI; *r* = *−*0.626, *P* = 0.001). In the low group, module 2 (represented by *Oscillospiraceae* NK4A214 group) was negatively correlated with CH_4_ emission (g/d; *r* = *−*0.673, *P* < 0.001; g/kg DMI; *r* = *−*0.546, *P* = 0.006), whereas module 7 (represented by *Epidinium*) was positively correlated with CH_4_ emission (g/d; *r* = 0.859, *P* < 0.001). In the med group, module 7 (represented by *Clostridium *sensu stricto 1) showed a negative correlation with CH_4_ emission (g/kg DMI; *r* = *−*0.624; *P* = 0.001). In the high group, module 5 (represented by *Eubacterium hallii* group) was negatively correlated with CH_4_ emission (g/kg DMI; *r* = *−*0.549, *P* = 0.005). The number of nodes and edges increased from the control (55 nodes, 76 edges) to the med group (59 nodes, 96 edges), but decreased in the high group (43 nodes, 38 edges) (Fig. 5E). Moreover, average degree and clustering coefficient were lowest in the high group (1.767 and 0.200) compared to the control (2.764 and 0.344). Based on Zi and Pi connectivity, microbial taxa were mostly classified as peripherals (taxa that interact less with others and are connected primarily within a module) across all groups such as control: (98.1%), low (86.3%), med (94.9%), and high (100%) with only one network hub and a few connectors identified. Although the majority of taxa were classified as peripherals based on Zi and Pi connectivity, several taxa showed distinct topological roles in the ST study (Fig S11A). In the control group, *Treponema* was identified as a connector taxon (taxa that connect different modules within a network**)**, whereas the low group featured multiple connector taxa, including F082, *Oscillospiraceae* NK4A214 group, *Anaerovorax*, *Succinivibrio*, and *Rikenellaceae* RC9 gut group, and *Succinivibrionaceae* UCG-002 was annotated as network hub. In the med group, *Lachnospiraceae* NK3A20 group, *Oscillospiraceae* NK4A214 group, and *Eubacterium hallii* group were classified as connectors. In contrast, all taxa detected in the high group were peripherals, with no connector, module hub, or network hub taxa observed.

**Fig. 6:**
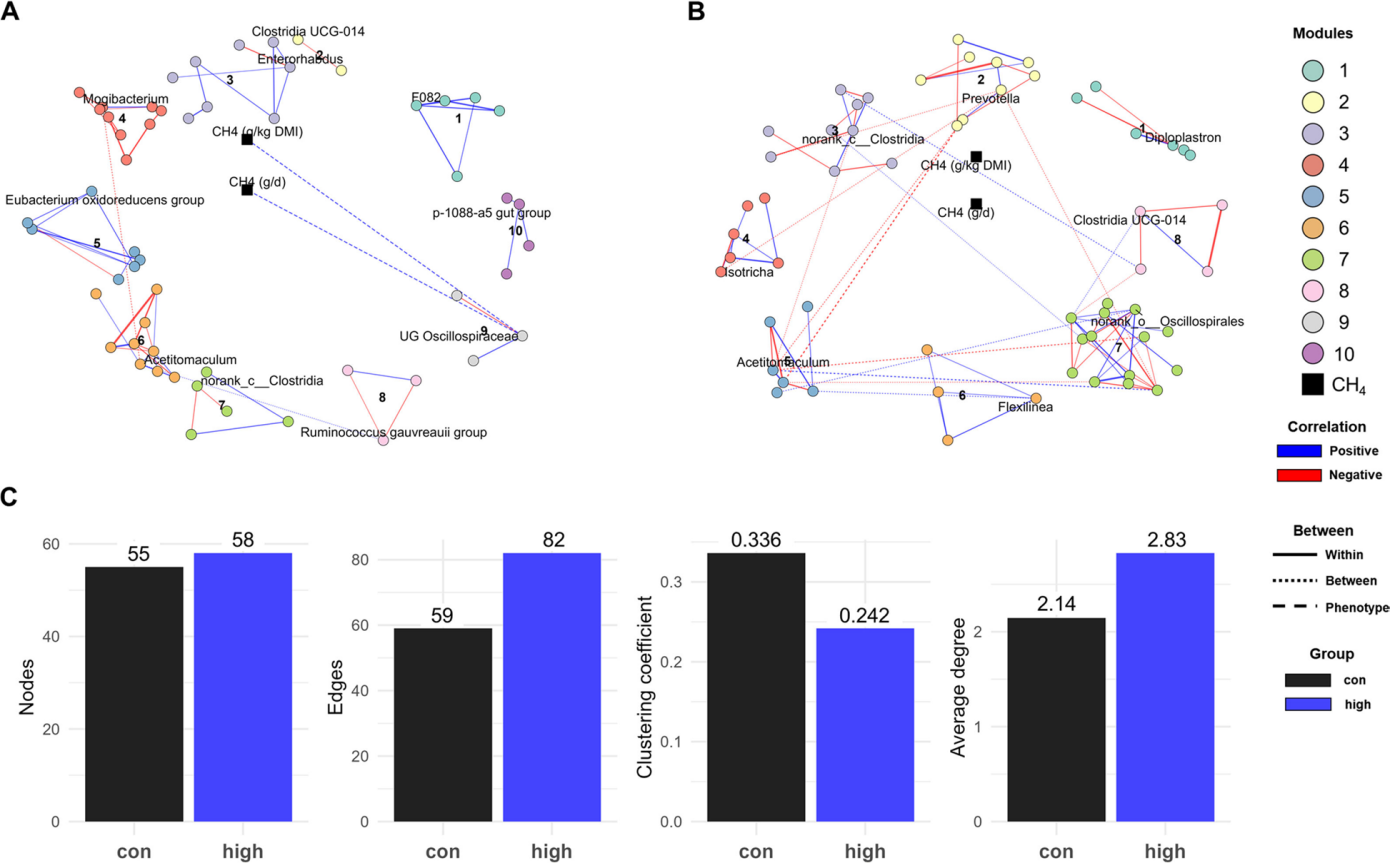
Co-occurrence networks of rumen microbial taxa after long-term 3-nitrooxypropanol (3-NOP) supplementation in beef cattle. **A **and** B **Microbial co-occurrence networks which were constructed based on Spearman’s correlation (|*r*| > 0.6, *P* < 0.05), and modular structures were identified. Node colors indicate module membership, with modules showing significant correlations to methane emission traits (CH_4_ g/d and CH_4_ g/kg DMI) connected to phenotype nodes (black). **C** Summarize the number of nodes, edges, clustering coefficient, and average degree for each treatment group. 3-NOP doses used in the long-term study were: control: 0 (black), high: 280 (blue), high-R*: 0 (green) mg/kg of DM. CH_4_: methane; DMI: dry matter intake

In the LT study, the co-occurrence networks showed high modularity in both groups, with a higher modularity in the control group (0.825) compared to the high group (0.643) (Table S11). As shown in Fig. 6A and B, co-occurrence networks comprising control (10) and high (8) modules were constructed, and the highest degree taxon in each module was annotated as a representative. In the control group, module 9 (represented by UG *Oscillospiraceae*) showed a positive correlation with CH_4_ emission (g/d; *r* = 0.715, *P* = 0.002; g/kg DMI; *r* = 0.715, *P* = 0.002), whereas none of the modules in the high group showed significant correlation with CH_4_ emission. The control showed less nodes (55 vs. 58) and edges (59 vs.82) than the high group, with a lower average degree (2.145 vs. 2.828) but higher clustering coefficient (0.336 vs. 0.242) (Fig. 6C ). Notably, there was a substantial difference in average betweenness, with the high 3-NOP group showing a much higher value (82.8) than the control group (10.6). Based on Zi and Pi connectivity, all microbial taxa in the control group were classified as peripherals (100%), while the high 3-NOP group showed a small proportion of connector taxa (3.45%) (Fig S11B).

**Fig. 7:**
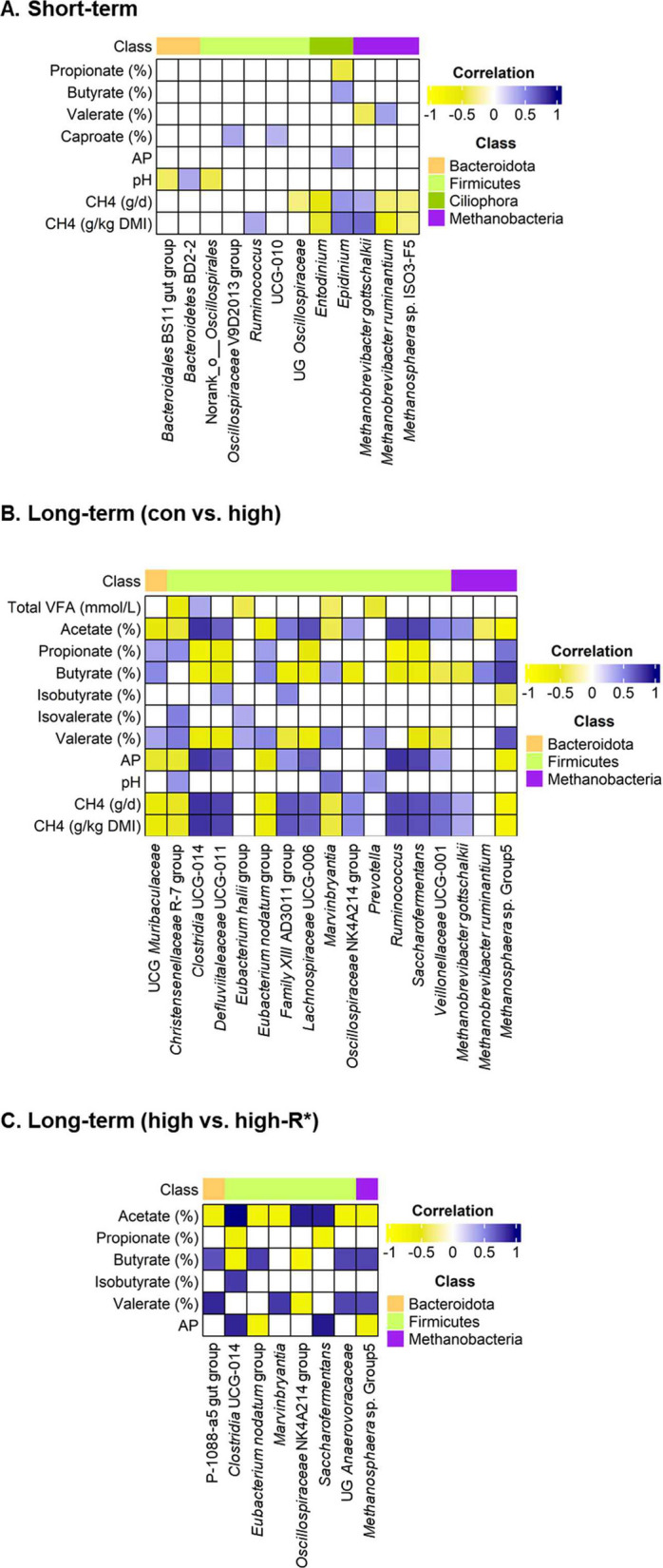
Spearman’s correlation analysis between rumen microbial taxa (bacteria, archaea, and protozoa) and animal phenotype profiles (including fermentation measurements and CH_4_ emission) after 3-nitrooxypropanol (3-NOP) supplementation in beef cattle for short-term (**A**) and long-term (**B** and **C**). For the long-term, **B** represents control vs. high 3-NOP groups, and **C** represents the high vs. recovery period (high-R*) groups. The class color indicates each phylum of the microbes. The intensity of the color indicates the degree of the correlation coefficient, based on the color key on the right side. The relative abundance of major phyla and genera (≥ 0.05% in more than 50% of animals) was analyzed for all individuals. Correlation coefficients (|*r*| ≥ 0.30) and significant (*P* < 0.05) correlations were selected to be shown on the plot. AP: acetate to propionate; R*: recovery period; control: control; CH_4_: methane; DMI: dry matter intake; UCG: uncultured genus-level; UG: unclassified genus-level; VFA: volatile fatty acid

### Correlation analysis among rumen microbial taxa, rumen fermentation characteristics, and methane emission

In the ST study, *Mbb*. *gottschalkii* showed positive correlation with CH_4_ emission (g/d: *r* = 0.363, *P* < 0.001; g/kg DMI: *r* = 0.611, *P* < 0.001), whereas *Mbb*. *ruminantium* and *Methanosphaera* sp. ISO3-F5 were negatively correlated with CH_4_ emission (g/d: *r* = *−*0.349, *P* = 0.001; g/kg DMI: *r* = *−*0.612, *P* < 0.001; g/d: *r* = *−*0.314, *P* = 0.002; g/kg DMI: *r* = *−*0.316, *P* = 0.002, respectively) (Fig. 7A). *Entodinium* showed negative correlation with CH_4_ emission (g/d: *r* = −0.511, *P* < 0.001; g/kg DMI: *r* = *−*0.476, *P* < 0.001), whereas *Epidninium* showed positive correlations (g/d: *r* = 0.445, *P* < 0.001; g/kg DMI: *r* = 0.586, *P* < 0.001).

Comparison between control vs. high groups in the LT study showed *Saccharofermentans* (*r* = 0.757, *P* < 0.001), *Ruminococcus* (*r* = 0.756, *P* < 0.001), and *Mbb. gottschalkii* (*r* = 0.460, *P* = 0.008) were positively correlated with molar proportion of acetate, while *Methanosphaera* sp. Group5 (*r* = *−*0.802, *P* < 0.001), *Mbb*. *ruminantium* (*r* = *−*0.383, *P* = 0.031), and *Eubacetrium nodatum* group (*r* = *−*0.614, *P* < 0.001) were negatively correlated with molar proportion of acetate (Fig. 7B). The CH_4_ emission variables were positively correlated with *Ruminococcus* (g/d: *r* = 0.734, *P* < 0.001; g/kg DMI: *r* = 0.734, *P* < 0.001), *Mbb. gottschalkii* (g/d: *r* = 0.372, *P* = 0.036; g/kg DMI: *r* = 0.372, *P* = 0.036), while negatively correlated with *Methanosphaera* sp. Group5 (g/d: *r* = *−*0.793, *P* < 0.001; g/kg DMI: *r* = *−*0.793, *P* < 0.001). Molar proportion of propionate showed positive correlation with *Methanosphaera* sp. Group5 (*r* = 0.584, *P* < 0.001) and negative correlations with *Clostridia* UCG-014 (*r* = *−*0.733, *P* < 0.001) and *Saccharofermentans* (*r* = *−*0.657, *P* < 0.001).

When comparing high and high-R* groups, molar proportion of acetate showed positive correlations with *Saccharofermentans* (*r* = 0.898, *P* = 0.002) and *Clostridia* UCG-014 (*r* = 0.999, *P* < 0.001) and negative correlations with *Marvinbryantia* (*r* = *−*0.762, *P* = 0.028), *Eubacterium nodatum* group (*r* = *−*0.786, *P* = 0.021) and *Methanosphaera* sp. Group5 (*r* = *−*0.762, *P* = 0.028) (Fig. 7C).

## Discussion

This study investigated bacterial, archaeal, and protozoal community composition and structure, together with their co-occurrence networks, to capture responses not addressed before, and is the first study to examine comprehensive microbial changes after 3-NOP withdrawal. Both ST and LT studies used similar basal diets consisting of 40% grain concentrate and 60% barley silage (DM basis, Table S1 and S2), and reported substantial CH_4_ reduction (g/kg DMI; ST: low: −4.39%, med: −9.30%, high: −33% and LT: −59%, respectively) due to 3-NOP supplementation. Recent meta-analysis of dietary 3-NOP supplementation of beef cattle diets has demonstrated that responses to 3-NOP are dose dependent: higher concentrations (101−329 mg/kg DM) result in greater reduction in CH_4_ emission (−6.82 g/kg DMI) compared to lower doses (35−100 mg/kg DM; −3.56 g/kg DMI) [41]. Additionally, the effectiveness of 3-NOP as a CH_4_ mitigation agent depends on how it is incorporated into the animal’s feed. Methane reduction is greater when 3-NOP is thoroughly mixed into the feed, as was done in the LT study, which helps synchronize 3-NOP delivery into the rumen with feed fermentation. In contrast, top-dressing 3-NOP (i.e., adding it on top of the feed without thorough mixing), as done in the ST study, causes the entire dose to be consumed at once, leading to a lack of sustained mitigation effects over a 24-h period. It is worth highlighting that CH_4_ measurements were conducted using respiration chambers, which provide the most accurate quantification method compared to other studies.

The present study demonstrates that 3-NOP decreased archaeal richness and altered community structure consistently across both ST and LT studies, confirming its impact on methanogenic community. In line with this, both ST and LT studies revealed consistent shifts in methanogen relative abundance after 3-NOP supplementation, with a decrease in *Mbb*. *gottschalkii* and increase in *Mbb*. *ruminantium* and *Methanosphaera* sp. These consistent patterns were further supported by the combined analysis across both studies, after correcting for study-specific batch effects, which revealed significant differences in methanogens between control and 3-NOP groups (Fig S8). Although study effects were significant, likely reflecting differences in supplementation method, dose, and study duration, 3-NOP still demonstrated significant effects on methanogens. This suggests that the impact of 3-NOP on methanogenic communities is robust and independent of study period. Moreover, Kittelmann et al. [42] reported a negative correlation between *Mbb*. *gottschalkii* and *Mbb*. *ruminantium* (*r* = *−*0.510, *P* = 0.023), which was more pronounced in our studies (ST: *r* = *−*0.992, *P* < 0.001; LT: *r* = *−*0.723, *P* < 0.001). This relationship aligns with previous findings that linked higher *Mbb*. *gottschalkii* relative abundance to increased CH_4_ emissions in both cattle [43] and sheep [44], while higher relative abundances of *Mbb*. *ruminantium* and *Methanosphaera* sp. were associated with lower CH_4_ emissions. Importantly, after withdrawal of 3-NOP, the microbial richness and evenness did not differ from control groups, with *Methanosphaera* sp. showing a significant decrease (*P* = 0.029) during the recovery period reaching the level after 3-NOP withdrawal, while the relative abundances of *Mbb*. *gottschalkii* and *Mbb*. *ruminantium* shifted back toward their initial levels, which were similar to those in the control group. These recovery patterns, along with no significant differences between control and 3-NOP recovery groups (control vs. high-R*), demonstrate that methanogenic communities can recover to their original composition after 3-NOP is withdrawn. The observed rebound in CH_4_ emissions after 3-NOP withdrawal can be explained by this community restructuring, particularly as recovered in the relative abundance of *Mbb*. *gottschalkii*, known as a major contributor to rumen methanogenesis. This recovery may be explained by the known ability of methanogens to reactivate MCR through a H_2_, ATP, and chaperone-dependent repair mechanism [9, 45, 46]. Although bacterial alpha diversity remained unchanged, beta diversity analysis revealed significant differences in community structure across 3-NOP supplementation in both ST and LT studies. Several bacterial taxa, including *Prevotella*, *Saccharofermentans*, and *Family XIII* AD3011 group, responded similarly in both studies, suggesting a consistent effect of 3-NOP on certain members in the bacterial communities. However, H_2_-producing bacteria such as *Clostridia* UCG-014 [47], *Ruminococcus* [48], and *Saccharofermentans* [49] decreased more prominently in the LT study, potentially due to differences in 3-NOP dose, feeding duration, and method. Since no studies have compared all three kingdoms of the rumen microbiota in both ST and LT 3-NOP supplementation, this study provides important value to our understanding of duration-driven microbial shifts and recovery.

The predicted functional shifts in rumen microbiota suggest potential common and distinct microbial adaptation strategies to 3-NOP supplementation depending on the duration. In both ST and LT studies, CH_4_ metabolism was consistently decreased, indicating that 3-NOP effectively inhibited methanogenesis by targeting MCR, a well-defined mode of action. Additionally, cysteine and methionine metabolism were decreased in both ST and LT studies. Recently, Takahashi and Tanaka III reported that L-cysteine contributes to H_2_ sulfide production, consuming H_2_ that would otherwise be utilized in methanogenesis via CO_2_ reduction [50]. We speculate that this decrease may reflect a lower demand for alternative H_2_ sinks due to H_2_ accumulation under suppressed methanogenesis. Notably, nitrogen metabolism was decreased in the ST study, while sulfur metabolism and D-glutamine and D-glutamate metabolism were decreased in the LT study, suggesting a duration-specific response is observed. Although 3-NOP supplementation affected rumen ammonia nitrogen (NH_3_-N) concentration in beef cattle (no effect: [10, 13, 14, 51]; decreased: [52]), no residual effects were observed on manure NH_3_ emissions during storage [53]. The observed decrease in nitrogen metabolism may reflect decreased proteolysis or enhanced NH_3_ uptake by rumen bacteria [54], as further supported by the ST-specific decrease in cysteine and methionine metabolism and the LT-specific reduction in D-glutamine and D-glutamate metabolism. The decrease of this pathway in the LT study may reflect a shift in carbon and nitrogen utilization under suppressed methanogenesis, as it is involved in microbial protein synthesis and competes for carbon that could otherwise contribute to CH_4_ production [55]. Some rumen *Clostridia* possess proteolytic and deaminative functions [56, 57], and the observed decrease in *Clostridia* UCG-014 in both ST and LT studies after 3-NOP supplementation may be associated with altered nitrogen metabolism. In addition, the ST study showed an increase in *Entodinium*, a protozoal genus known to play a key role in nitrogen turnover [58], whereas the LT study showed a decrease in *Ruminococcus* and the *Ruminococcus gauvreauii* group [59], both of which are involved in protein degradation and nitrogen recycling. These taxonomic shifts may reflect functional adjustments in nitrogen metabolism under different durations of 3-NOP supplementation. However, our phenotype data showed no significant differences in NH_3_-N concentrations in either the ST or LT studies, suggesting that while 3-NOP may transiently influence specific nitrogen-related pathways, it does not appear to substantially disrupt overall nitrogen utilization within the rumen ecosystem.

Furthermore, VFA metabolism differed between the ST and LT studies, with decreased propionate metabolism in the ST study and decreased butanoate metabolism in the LT study. However, phenotype data showed increased molar proportions of both VFAs [13, 14], along with a higher relative abundance of associated microbial taxa such as *Prevotella* and *Succiniclasticum*. Fumarate reductase (EC:1.3.5.4), a key enzyme involved in propionate production via fumarate reduction, was increased in both the ST and LT studies based on PICRUSt2 functional prediction. It is plausible that the increased relative abundance of *Succiniclasticum* in the 3-NOP groups compared to the control group, and the presence of *Succinivibrionaceae* UCG-002 as the highest degree taxon in multiple modules support this metabolic shift. In the ST study, *Succinivibrionaceae* UCG-002 is likely cooperating (co-occurred) with *Fibrobacter* and several *Ruminococcaceae* members, which are known to possess fumarate-reducing capabilities [60, 61]. This co-occurrence indicates their collective contribution to the rumen metabolic network, ultimately enhancing propionate production as an alternative H_2_ sink. In the LT study, *Prevotella* was identified as a connector taxon with the highest degree in a propionate-associated module, which also included *Prevotellaceae* UCG-001 and *Muribaculaceae* [62]. The increased relative abundance and central role of *Prevotella* may have supported microbial interactions that promote propionate production. However, the inconsistent result of decreased propionate metabolism may reflect a limitation of functional prediction based on 16S rRNA gene sequences by both CowPI and PICRUSt2-SC. It is possible that the gene-level relative abundance of enzymes involved in propionate synthesis, such as those encoding fumarate reductase, may vary across microbial taxa and were not consistently predicted. Previously, we conducted metagenomic analysis of the ST and LT studies, in which the relative abundance of propionate-related genes showed inconsistent patterns, with *LDH* (L-lactate dehydrogenase) and *sucD* (succinyl-CoA synthetase) decreased and *ACADS* (acyl-CoA dehydrogenase) increased, making it difficult to draw clear biological conclusions. Therefore, metatranscriptomic analysis is required to determine the actual activity of these genes and to better understand the functional responses related to propionate metabolism in the rumen microbiota under 3-NOP supplementation. Notably, the LT study showed that the increased relative abundance of the pectin degradation pathway (M00081; Coeff: 0.004, *Q* = 0.002) suggests enhanced methanol production, as pectin is a major precursor for methanol release in the rumen. Pectin is degraded by microorganisms with pectinolytic activity, such as *Fibrobacter* [63], *Butyrivibrio* [64], *Prevotella* [64], *Treponema* [65], and *Entodinium* [58]. In the present study, both *Prevotella* and *Entodinium* were more abundant in the 3-NOP supplemented groups than in the control group, potentially contributing to elevated methanol production via enhanced pectin degradation, as previously described by Kelly et al. [64]. This could partly explain the observed increase in *Methanosphaera* sp., a methylotrophic methanogen that utilizes methanol as its primary substrate.

When comparing the high and high-R* groups, we observed notable differences in H_2_ producers such as *Clostridia* UCG-014 and *Saccharofermentans*, which decreased significantly in the high group but returned to control levels in the high-R* group. Greening et al. [66] reported that both *Clostridia* and *Saccharofermentans* account for a large proportion of hydrogenases in the rumen and inferred that fermentative *Clostridia* are predominant in this functional role. Notably, *Saccharofermentans* was found to be a major source of hydrogenases, accounting for 9.2% of group A3 [FeFe]-hydrogenase transcripts, which play an important role in ruminal H_2_ metabolism and energy conservation [66, 67]. However, the decrease in *Clostridia* UCG-014 and *Saccharofermentans* relative abundance during 3-NOP supplementation suggests that despite having fermentative hydrogenase capability, its metabolic contribution to ruminal H_2_ metabolism might be decreased. These findings indicate that the impact of 3-NOP on rumen H_2_ metabolism indirectly affects various H_2_ producing bacteria through its direct inhibition of methanogenesis. In the high group, *Clostridia* UCG-014 was identified as the highest degree taxon in module 8, which also included *Methanosphaera* sp. Group5. These genera showed a strong negative correlation (*r* = *−*0.676, *P* = 0.004), possibly reflecting altered H_2_ dynamics under 3-NOP supplementation.

A previous study reported that methanogen species have distinct relationships with bacteria, such as *Mbb*. *gottschalkii* is associated with *Ruminococcaceae* and *Mbb*. *ruminantium* is associated with *Fibrobacteraceae* [42]. As primary cellulose degraders, *Ruminococcus* sp. produce large amounts of H_2_ [68], while *Fibrobacter* sp. produces formate [69], both of which serve as substrates for methanogens. Consistent with this association, *Ruminococcus* sp. and *Mbb*. *gottschalkii* showed concurrent decreases and positive correlation [ST: *r* = 0.354, *P* < 0.001; LT (control vs. high): *r* = 0.521, *P* = 0.002] with 3-NOP supplementation in both studies.

From a network perspective, our analysis suggested that 3-NOP supplementation and its dosage levels drove distinct structural and topological changes in the microbial communities, compared to the control group, in both ST and LT studies. In the ST study, the high group exhibited the lowest network metrics (nodes, edges, average degree, clustering coefficient), indicating a sparsely connected microbial network. Notably, all taxa were classified as peripherals, suggesting the absence of both central and connector taxa, reflecting significantly decreased microbial interactions at the highest 3-NOP dose. In contrast, the low and med groups displayed more diverse topological roles, including several connector taxa, and the low group uniquely featured a network hub identified as *Succinivibrionaceae* UCG-002. In contrast to the ST study, the LT study revealed that the high group showed a more connected microbial network than the control, with increased numbers of edges, a higher average degree, and notably elevated betweenness centrality. While all taxa in the control group were classified as peripherals, the high group included two connector taxa including, *Prevotella* and *Acetitomaculum*, which may play central roles in mediating interactions across modules. These contrasting patterns between ST and LT studies suggest that the impact of 3-NOP on the rumen microbial network is both dose- and duration-dependent. While high-dose supplementation in the ST study simplified the microbial network and reduced microbial interactions, low doses preserved key connector taxa and network hubs. In contrast, the LT study showed that long-term supplementation promoted the emergence of key connector taxa, supporting a more structured microbial network. The discrepancy in microbial responses between ST and LT studies might be attributed to differences in treatment duration and dose levels [22]. In the ST study, feed was offered ad libitum, whereas in the LT study feed intake was restricted to 65% of ad libitum intake, and therefore, animals fed the highest dose in the ST study consumed 2.79 g/d of 3-NOP compared to 2.00 g/d in the LT study.

Protozoa play a crucial role in rumen methanogenesis, with methanogens attached to their surface generating H_2_ via hydrogenosomes in a synergistic relationship [70]. Considering that protozoa-associated methanogens (PAMs) are predominantly *Mbb*. sp. (79%) [71], the decrease in *Mbb*. *gottschalkii* relative abundance with 3-NOP supplementation suggests that PAMs were also decreased. While 3-NOP supplementation did not significantly affect the total protozoa abundance in the ST study, it altered the protozoal composition, with *Entodinium* relative abundance increased and *Epidnium* relative abundance decreased with the high-dose of 3-NOP. Most rumen protozoa produce H_2_ through hydrogenosomes, but *Entodinium* lacks these hydrogenosomes and instead depends on mitosomes and Fe-hydrogenases [56, 57], which may support H_2_ production beneficial to methanogenic endosymbionts. *Entodinium* is the most predominant rumen ciliate species that preferentially utilizes starch [72], and is reported to be less involved in promoting methanogenesis compared to *Isotricha* [73], which may contribute to reduced CH_4_ production after 3-NOP supplementation. Its starch-utilizing characteristics could support propionate-producing bacteria as suggested by an in vitro study [74]. The starch-utilizing characteristics of *Entodinium* and its potential to support propionate-producing bacteria was reflected in our results with increased relative abundance of *Entodinium* after 3-NOP supplementation and increased relative abundance of propionate-producing bacteria (*Prevotella*, *Succiniclasticum*, and *Anaerovibrio*). Although direct correlations between *Entodinium* and these bacteria were not observed, *Entodinium* showed negative correlations with CH_4_ emissions (g/d: *r* = *−*0.511, *P* < 0.001; g/kg DMI: *r* = *−*0.476, *P* < 0.001) and *Epidinium* (*r* = *− *0.622, *P* < 0.001). While few beef cattle studies that evaluated 3-NOP report effects on protozoa, a recent study (200 mg/kg of DM for 28-d) showed significant reduction in *Mbb*. sp. but no changes in *Entodinium* relative abundance [75], unlike our findings where both were affected. These contrasting results may be attributed to differences in diet composition or 3-NOP dose levels suggesting that protozoa and methanogen relationships with 3-NOP supplementation may not be consistent and warrant further investigation in beef cattle. Additionally, *Epidinium* is known to harbor intra- and extracellular methanogenic archaea [76] and contributes to increased CH_4_ production in the rumen [77]. This genus has hydrogenosomes, which enables symbiotic interactions with hydrogenotrophic methanogens as suggested by Park et al. [78]. Notably, *Epidinium* has been found to dominate A1 hydrogenase reads, further supporting its role in H_2_ metabolism and potential interactions with hydrogenotrophic methanogens [66]. Although the exact mechanism by which 3-NOP supplementation affected *Epidinium* remains unclear, the decreased relative abundance may be associated with the decrease in *Mbb*. *gottschalkii* relative abundance. This is supported by the observation that *Epidinium* showed a positive correlation with *Mbb*. *gottschalkii* (*r* = 0.358, *P* < 0.001) and CH_4_ emission (g/d: *r* = 0.445, *P* < 0.001; g/kg DMI: *r* = 0.586, *P* < 0.001). While 3-NOP supplementation decreased *Epidinium* relative abundance in the ST study, the LT study revealed an increase in total protozoa absolute abundance without significant differences in genera composition, suggesting that prolonged exposure to 3-NOP may have created a rumen environment that resulted in higher protozoal relative abundance rather than shifting the dominance of specific genera. During the recovery period, no differences in *Epidinium* relative abundance between high and high-R* groups (3-NOP supplementation vs. withdrawal) indicates that withdrawal of 3-NOP had no effect on these protozoa. Consistently, estimated absolute abundance also showed no significant difference, supporting this result. Although 3-NOP effectively reduces CH_4_ emissions, the LT study with recovery period demonstrated this effect is temporary, with emissions returning to normal (control) levels after withdrawal. These results are interpreted to mean that continuous supplementation is necessary for sustained CH_4_ reduction. The finding that microbial composition and fermentation patterns returned to normal or near-normal levels after withdrawal of 3-NOP from the diet demonstrates that the effects on the rumen environment are not permanent.

The limitation of this study is the use of 454 pyrosequencing technology, which is not widely used recently. Although the sequencing depth of our 454 pyrosequencing is not as high as the currently prevalent Illumina platforms for amplicon sequencing, the Good's coverage reached 99.9% for bacterial, archaeal, and protozoal communities, respectively, demonstrating adequate coverage of the microbial community. While lower sequencing depth may reduce sensitivity for rare taxa and slightly constrain resolution in alpha-diversity, beta-diversity, and network analyses, the dominant community members were consistently captured, and major ecological patterns remained robust. Moreover, similar findings of richness and evenness have been reported in rumen studies using Roche 454 pyrosequencing [79–82], suggesting that the present study is within the expected range of microbial diversity assessments despite the limitations of the sequencing technology. Furthermore, power analysis confirmed that the sample size (*n* = 8) provided sufficient statistical power (> 0.99 at α = 0.05) to detect treatment effects in both ST and LT studies.

**Fig. 8:**
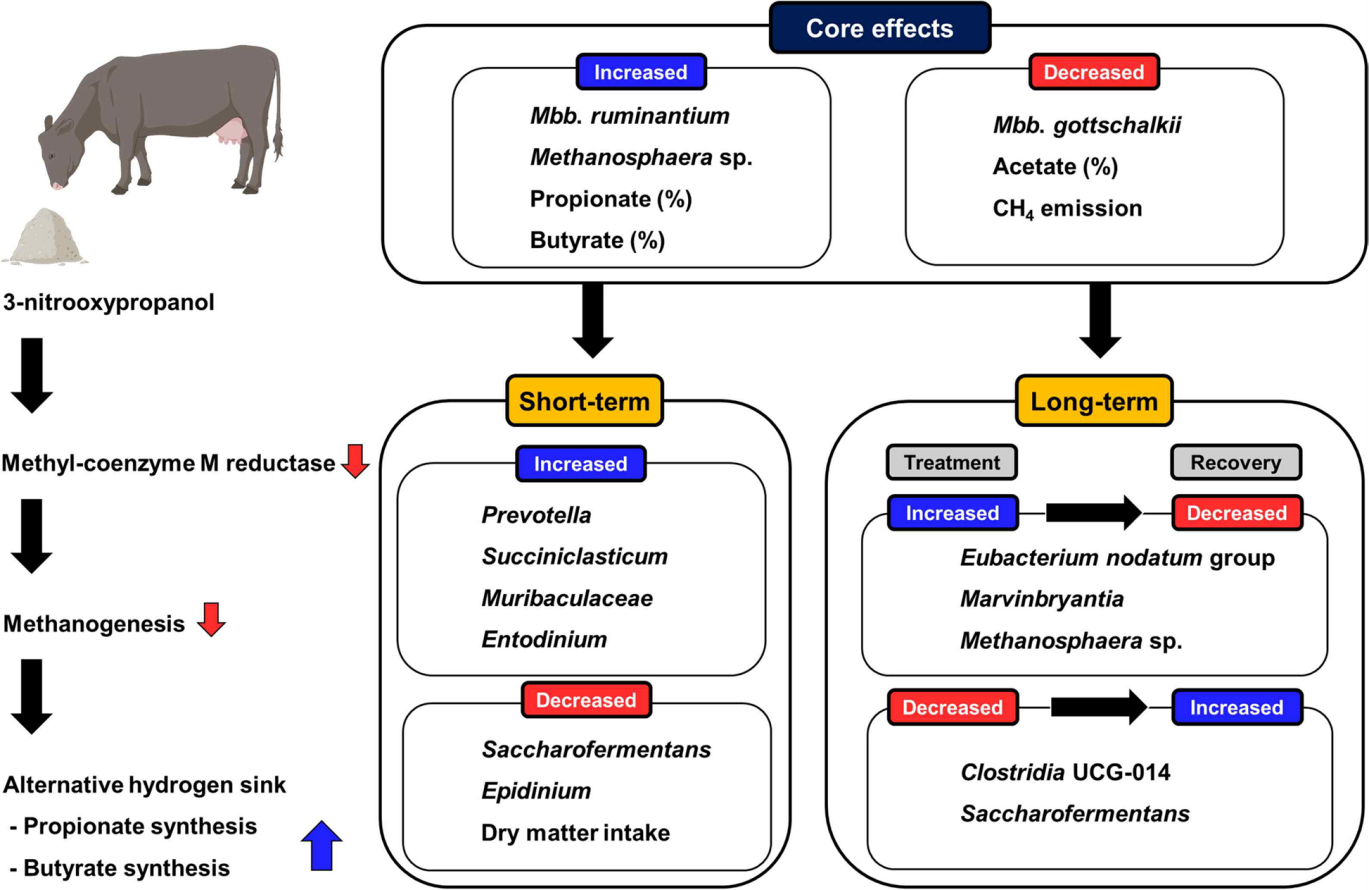
Summarized model after 3-nitrooxypropanol (3-NOP) supplementation on rumen microbial composition and fermentation in short-term and long-term studies. Core effects refer to shared impacts observed in both short- and long-term 3-NOP supplementation studies. Directional changes are shown in blue (increased) and red (decreased). UCG: uncultured genus-level; CH_4_: methane; VFA: volatile fatty acid; *Mbb.*: *methanobrevibacter*

The effects of 3-NOP supplementation on rumen microbial composition are summarized in Fig. 8. Methanogen composition changed in both studies, with decreased *Mbb*. *gottschalkii* and increased *Mbb*. *ruminantium* and *Methanosphaera* sp., reflecting their responses to 3-NOP supplementation. The bacterial and protozoal responses varied between the ST and LT studies such that ST supplementation enhanced propionate-producing bacteria (*Prevotella*, *Succiniclasticum*) and altered protozoal composition (increased *Entodinium*, decreased *Epidinium*), while LT supplementation primarily led to decrease acetate-producing bacteria (*Ruminococcus*, *Ruminococcus gauvreauii* group, and *Saccharofermentans*) but increase total protozoa absolute abundance. These differences potentially stemmed from variations in 3-NOP dose levels and duration of supplementation. In addition, microbial network analysis showed that both the dose and duration of 3-NOP supplementation affected microbial community structure. The high-dose group in the ST study showed simplified networks consisting only of peripheral taxa, while the high group in the LT study showed more modular and connected networks with several connector taxa. Notably, several microbial groups (*Eubacterium nodatum* group, *Marvinbryantia*, *Saccharofermentans*, *Clostridia* UCG-014, and *Methanosphaera* sp.) demonstrated dynamic responses to 3-NOP presence and absence, as their abundances changed during supplementation and returned to normal levels after withdrawal, suggesting these are key microbes warranting further investigation of their capabilities under 3-NOP supplementation.

## Conclusions

This study demonstrates that microbial responses to 3-NOP supplementation differ distinctly between ST and LT applications in beef cattle. By capturing microbial responses across bacteria, archaea, and protozoa over different supplementation durations, this study contributes new understanding to the temporal dynamics of 3-NOP effects in the rumen. Withdrawal of 3-NOP led to recovery of methanogen communities, as *Mbb*. *gottschalkii* and *Mbb*. *ruminantium* returned to control levels while *Methanosphaera* sp. Group5 decreased. This recovery was accompanied by a rebound in CH_4_ emissions, demonstrating that the effects of 3-NOP were reversible. In addition, the identification of specific bacterial groups with reversible responses, such as *Clostridia* UCG-014 and *Eubacterium nodatum* group, provided further insight into rumen adaptation patterns. Importantly, changes in methanogen community structure were consistently observed regardless of supplementation duration, whereas bacterial and protozoal responses were duration-dependent. These patterns remained consistent after batch effect correction in the combined analysis, confirming that the impact of 3-NOP on methanogens is robust across different study conditions. Predicted functional analysis suggested that CH_4_ metabolism and nitrogen-related metabolism were decreased. Network analysis showed that 3-NOP supplementation altered the microbial community structure, with simplified networks under ST high-dose treatment and more connected, modular networks in LT supplementation. However, we acknowledge several confounding factors in this study, including variations in feeding regimes (restricted vs. ad libitum), 3-NOP feeding methods (top-dressing vs. mixed in the feed) and doses, time points, and periods, which warrant further investigation under more controlled conditions. These findings highlight the importance of considering both supplementation duration and feeding management for optimal CH_4_ mitigation strategies in beef cattle.

## Supplementary Information

Additional file 1: Table S1. Ingredient and chemical composition of the basal diet in the short-term study. Table S2. Ingredient and chemical composition of the basal diet in the long-term study.
Additional file 2: Table S3. Pairwise comparisons of beta diversity of bacterial, archaeal, and protozoal communities after short-term 3-NOP supplementation in beef cattle. Table S4. Pairwise comparisons of beta diversity of bacterial, archaeal, and protozoal communities after long-term 3-NOP supplementation in beef cattle. Table S5. Effects of short-term 3-NOP supplementation on rumen microbial taxa (bacteria, archaea, and protozoa) based on relative and estimated absolute abundances. Table S6. Effects of long-term 3-NOP supplementation on rumen microbial taxa (bacteria, archaea, and protozoa) based on relative and estimated absolute abundances. Table S7. Effects of long-term 3-NOP supplementation and withdrawal on rumen microbial taxa (bacteria, archaea, and protozoa) based on relative and estimated absolute abundances. Table S8. Effects of short-term 3-NOP supplementation on predicted rumen functions based on CowPI. Table S9. Effects of long-term 3-NOP supplementation on predicted rumen functions based on CowPI. Table S10. Summary of microbial co-occurrence network metrics in the short-term 3-NOP supplementation. Table S11. Summary of microbial co-occurrence network metrics in the long-term 3-NOP supplementation.

Additional file 3: Fig. S1. Rarefaction curves of bacterial, archaeal, and protozoal communities after short- and long-term 3-NOP supplementation in beef cattle. Fig. S2. Comparison of alpha (Shannon index) and beta (Bray–Curtis dissimilarity) diversities of bacterial, archaeal, and protozoal communities in short-term (ST) and long-term (LT) periods. Fig. S3. Venn diagrams showing the genera of rumen microbes shared between and unique to short- and long-term studies. Fig. S4. Comparison of rumen microbial taxa after short-term 3-NOP supplementation in beef cattle. Fig. S5. Comparison of rumen microbial taxa between control and high groups after long-term 3-NOP supplementation in beef cattle. Fig. S6. Comparison of rumen microbial taxa between control and high-R* (recovery) groups after long-term 3-NOP supplementation in beef cattle. Fig. S7. Comparison of rumen microbial taxa between high and high-R* (recovery) groups after long-term 3-NOP supplementation in beef cattle. Fig. S8. Comparison of rumen microbial taxa after 3-NOP supplementation in beef cattle in short- and long-term periods. Fig. S9. Differentially abundant predicted microbial functions associated with methane metabolism after short-term 3-NOP supplementation in beef cattle. Fig. S10. Differentially abundant predicted microbial functions associated with methane metabolism after long-term 3-NOP supplementation in beef cattle. Fig. S11. Topological roles of rumen microbial taxa derived from co-occurrence networks after short-term and long-term 3-NOP supplementation.

## Data Availability

Pyrosequencing data of rumen microbiota gene sequences are available at the National Center for Biotechnology Information (NCBI) under project numbers PRJNA1150225 (short-term) and PRJNA1150246 (long-term).

## Abbreviations

3-NOP: 3-Nitrooxypropnaol
CH_4_: Methane
DM: Dry matter
H_2_: Hydrogen
LT: Long-term
*Mbb.*: *Methanobrevibacter*
MCR: Methyl coenzyme M reductase
NH_3_: Ammonia
NH_3_-N: Ammonia nitrogen
R*: Recovery
ST: Short-term
VFA: Volatile fatty acid
