# Supplementary material for: Short- and long-term dietary supplementation as well as withdrawal of the enteric methane inhibitor 3-nitrooxypropanol reveal distinct effects on the rumen microbial community
Source: J Anim Sci Biotechnol. 2025 Dec 1;16:162. doi: 10.1186/s40104-025-01291-w (PMC12667093; doi:10.1186/s40104-025-01291-w)
Supplement: Supplementary file 1 — Additional file 1: Table S1. Ingredient and chemical composition of the basal diet in the short-term study. Table S2. Ingredient and chemical composition of the basal diet in the long-term study. Additional file 2: Table S3. Pairwise comparisons of beta diversity of bacterial, archaeal, and protozoal communities after short-term 3-NOP supplementation in beef cattle. Table S4. Pairwise comparisons of beta diversity of bacterial, archaeal, and protozoal communities after long-term 3-NOP supplementation in beef cattle. Table S5. Effects of short-term 3-NOP supplementation on rumen microbial taxa (bacteria, archaea, and protozoa) based on relative and estimated absolute abundances. Table S6. Effects of long-term 3-NOP supplementation on rumen microbial taxa (bacteria, archaea, and protozoa) based on relative and estimated absolute abundances. Table S7. Effects of long-term 3-NOP supplementation and withdrawal on rumen microbial taxa (bacteria, archaea, and protozoa) based on relative and estimated absolute abundances. Table S8. Effects of short-term 3-NOP supplementation on predicted rumen functions based on CowPI. Table S9. Effects of long-term 3-NOP supplementation on predicted rumen functions based on CowPI. Table S10. Summary of microbial co-occurrence network metrics in the short-term 3-NOP supplementation. Table S11. Summary of microbial co-occurrence network metrics in the long-term 3-NOP supplementation. [file 40104_2025_1291_MOESM1_ESM.docx]

**Table S1** Ingredient and chemical composition of the basal diet in short-term study*

| **Item** | **% of DM** |
| --- | --- |
| Ingredient^1,2^ |  |
| Barley silage^3^ | 60 |
| Barley grain, dry rolled | 35 |
| Barley grain, ground | 2.688 |
| Calcium carbonate | 1.374 |
| Canola meal | 0.500 |
| Salt | 0.158 |
| Urea | 0.110 |
| Molasses, dried | 0.108 |
| LRC feedlot vitamin-mineral premix^4^ | 0.055 |
| Vitamin E (500,000 IU/kg) | 0.004 |
| Flavoring agent | 0.003 |
| Chemical composition^5^ |  |
| DM | 46.7 ± 2.05 |
| OM, % of DM | 92.9 ± 0.43 |
| CP, % of DM | 11.7 ± 0.25 |
| NDF, % of DM | 37.6 ± 1.31 |
| ADF, % of DM | 20.6 ± 0.55 |
| Starch, % of DM | 31.8 ± 1.03 |
| Fat, % of DM | 2.7 ± 0.19 |

^1^All ingredients except barley silage and dry-rolled barley grain were provided as part of a pelleted supplement.

^2^Each heifer received 2.69 mg/d of melengesterol acetate as a pellet to suppress estrous activity. Pellet contained MGA-100 premix (Pfizer Canada Inc., Kirkland, QC, Canada), 0.45%; ground barley grain, 95.99%; dried molasses, 2.51%; and flavoring agent, 0.05% (DM basis). It was fed at 600 g/animal daily (as-is basis).

^3^Composition: DM, 34.5%; CP, 11.4%; NDF, 49.7%; ADF, 32.3%; starch, 16.1%; and GE, 5.6 Mcal/kg.

^4^Feedlot vitamin-mineral premix contained CaCO_3_, 35.01%; CuSO_4_, 10.37%; ZnSO_4_, 28.23%; Ethylenediamine dihydriodide (80% concentration), 0.15%; selenium 1% (10,000 mg Se/kg), 5.01%; CoSO4, 0.1%; MnSO_4_, 14.54%; vitamin A (500,000,000 IU/kg), 1.71%; vitamin D (500,000,000 IU/ kg), 0.17%; and vitamin E (500,000 IU/kg), 4.7%.

^5^Mean ± SD; *n* = 4

*This table was reproduced from Romero-Pérez et al. [13].

**Table S2** Ingredient and chemical composition of the basal diet in long-term study*

| **Item** | **% of DM** |
| --- | --- |
| Ingredient^1^ |  |
| Barley silage^2^ | 60 |
| Barley grain, dry rolled | 35 |
| Barley grain, ground | 2.688 |
| Calcium carbonate | 1.374 |
| Canola meal | 0.500 |
| Salt | 0.158 |
| Urea | 0.110 |
| Molasses, dried | 0.108 |
| LRC feedlot vitamin-mineral premix^3^ | 0.055 |
| Vitamin E (500,000 IU/kg) | 0.004 |
| Flavoring agent | 0.003 |
| Chemical composition^4^ |  |
| DM | 53.6 ± 2.18 |
| OM, % of DM | 92.5 ± 0.24 |
| CP, % of DM | 11.3 ± 0.63 |
| NDF, % of DM | 38.6 ± 1.35 |
| ADF, % of DM | 22.4 ± 1.72 |
| Starch, % of DM | 33.8 ± 0.57 |
| Fat, % of DM | 1.85 ± 0.13 |
| GE, Mcal/kg | 4.9 0.53 |

^1^All ingredients except barley silage and dry-rolled barley grain were provided as part of a pelleted supplement.

^2^Composition: 42.4% DM, 10.5% CP, 49.9% NDF, and 33.1% ADF.

^3^Feedlot vitamin–mineral premix contained 35.01% CaCO_3_, 10.37% CuSO_4_, 28.23% ZnSO_4_, 0.15% ethylenediamine dihydriodide (80% concentration), 5.01% selenium 1% (10,000 mg Se/kg), 0.1% CoSO_4_, 14.54% MnSO_4_, 1.71% vitamin A (500,000,000 IU/kg), 0.17% vitamin D (500,000,000 IU/kg), and 4.7% vitamin E (500,000 IU/kg).

^4^Mean ± SD; *n* = 6

*This table was reproduced from Romero-Pérez et al. [14].
