# Supplementary material for: Short- and long-term dietary supplementation as well as withdrawal of the enteric methane inhibitor 3-nitrooxypropanol reveal distinct effects on the rumen microbial community
Source: J Anim Sci Biotechnol. 2025 Dec 1;16:162. doi: 10.1186/s40104-025-01291-w (PMC12667093; doi:10.1186/s40104-025-01291-w)
Supplement: Supplementary file 2 — Additional file 3: Fig. S1. Rarefaction curves of bacterial, archaeal, and protozoal communities after short- and long-term 3-NOP supplementation in beef cattle. Fig. S2. Comparison of alpha (Shannon index) and beta (Bray–Curtis dissimilarity) diversities of bacterial, archaeal, and protozoal communities in short-term (ST) and long-term (LT) periods. Fig. S3. Venn diagrams showing the genera of rumen microbes shared between and unique to short- and long-term studies. Fig. S4. Comparison of rumen microbial taxa after short-term 3-NOP supplementation in beef cattle. Fig. S5. Comparison of rumen microbial taxa between control and high groups after long-term 3-NOP supplementation in beef cattle. Fig. S6. Comparison of rumen microbial taxa between control and high-R* (recovery) groups after long-term 3-NOP supplementation in beef cattle. Fig. S7. Comparison of rumen microbial taxa between high and high-R* (recovery) groups after long-term 3-NOP supplementation in beef cattle. Fig. S8. Comparison of rumen microbial taxa after 3-NOP supplementation in beef cattle in short- and long-term periods. Fig. S9. Differentially abundant predicted microbial functions associated with methane metabolism after short-term 3-NOP supplementation in beef cattle. Fig. S10. Differentially abundant predicted microbial functions associated with methane metabolism after long-term 3-NOP supplementation in beef cattle. Fig. S11. Topological roles of rumen microbial taxa derived from co-occurrence networks after short-term and long-term 3-NOP supplementation. [file 40104_2025_1291_MOESM2_ESM.zip › 40104_2025_1291/Choi et al. R2 Additional file 2, Figure S2.pdf]

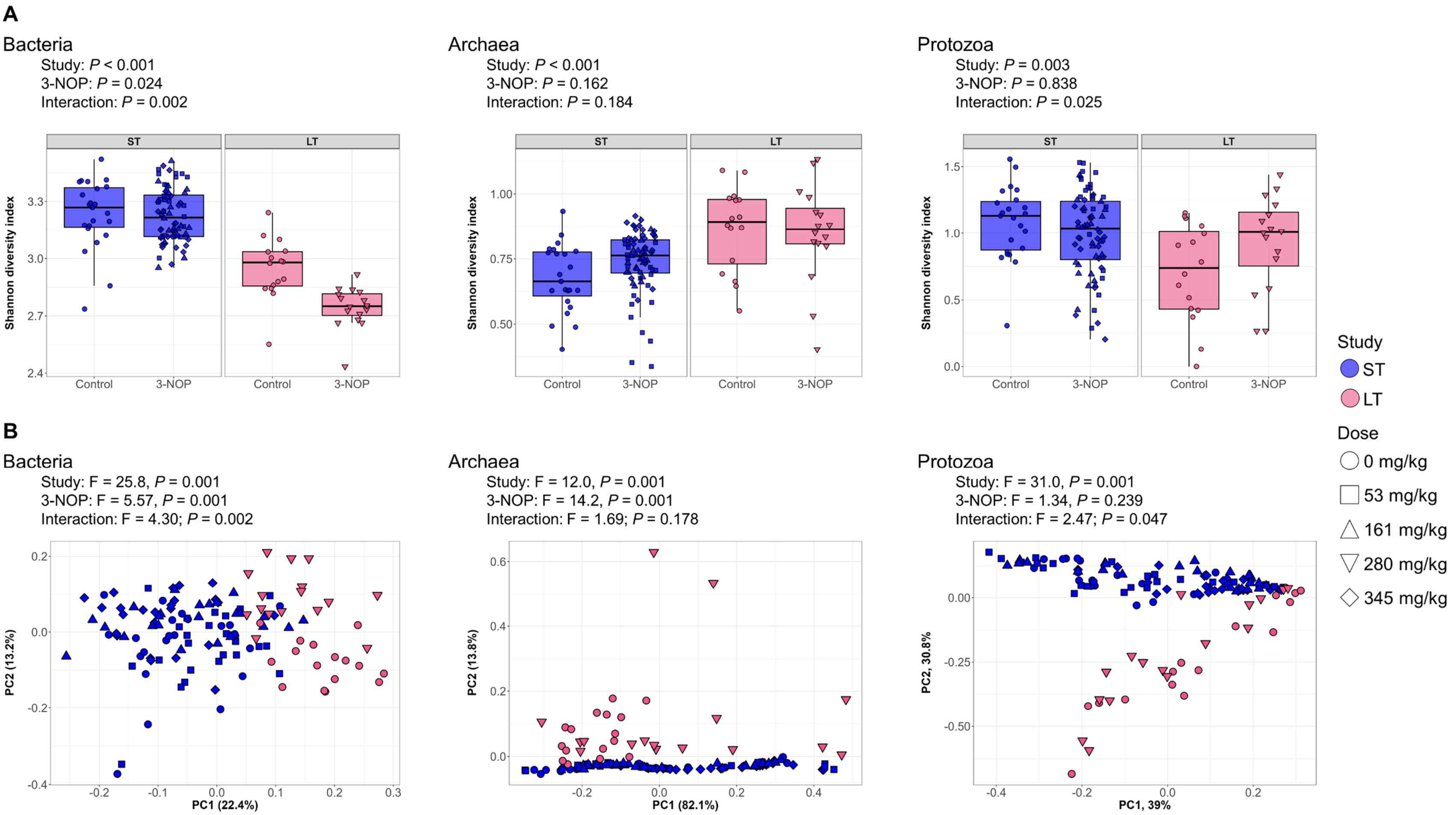
A
Bacteria
Study: P < 0.001
3-NOP: P = 0.024
Interaction: P = 0.002
Archaea
Study: P < 0.001
3-NOP: P = 0.162
Interaction: P = 0.184
Protozoa
Study: P = 0.003
3-NOP: P = 0.838
Interaction: P = 0.025
ST
LT
Shannon diversity index
Control
3-NOP
B
Bacteria
Study: F = 25.8, P = 0.001
3-NOP: F = 5.57, P = 0.001
Interaction: F = 4.30; P = 0.002
PC1 (22.4%)
PC2 (13.2%)
Archaea
Study: F = 12.0, P = 0.001
3-NOP: F = 14.2, P = 0.001
Interaction: F = 1.69; P = 0.178
PC1 (82.1%)
PC2 (13.8%)
Protozoa
Study: F = 31.0, P = 0.001
3-NOP: F = 1.34, P = 0.239
Interaction: F = 2.47; P = 0.047
PC1, 39%
PC2, 30.8%
Study
ST
LT
Dose
0 mg/kg
53 mg/kg
161 mg/kg
280 mg/kg
345 mg/kg
