# Supplementary material for: Short- and long-term dietary supplementation as well as withdrawal of the enteric methane inhibitor 3-nitrooxypropanol reveal distinct effects on the rumen microbial community
Source: J Anim Sci Biotechnol. 2025 Dec 1;16:162. doi: 10.1186/s40104-025-01291-w (PMC12667093; doi:10.1186/s40104-025-01291-w)
Supplement: Supplementary file 2 — Additional file 3: Fig. S1. Rarefaction curves of bacterial, archaeal, and protozoal communities after short- and long-term 3-NOP supplementation in beef cattle. Fig. S2. Comparison of alpha (Shannon index) and beta (Bray–Curtis dissimilarity) diversities of bacterial, archaeal, and protozoal communities in short-term (ST) and long-term (LT) periods. Fig. S3. Venn diagrams showing the genera of rumen microbes shared between and unique to short- and long-term studies. Fig. S4. Comparison of rumen microbial taxa after short-term 3-NOP supplementation in beef cattle. Fig. S5. Comparison of rumen microbial taxa between control and high groups after long-term 3-NOP supplementation in beef cattle. Fig. S6. Comparison of rumen microbial taxa between control and high-R* (recovery) groups after long-term 3-NOP supplementation in beef cattle. Fig. S7. Comparison of rumen microbial taxa between high and high-R* (recovery) groups after long-term 3-NOP supplementation in beef cattle. Fig. S8. Comparison of rumen microbial taxa after 3-NOP supplementation in beef cattle in short- and long-term periods. Fig. S9. Differentially abundant predicted microbial functions associated with methane metabolism after short-term 3-NOP supplementation in beef cattle. Fig. S10. Differentially abundant predicted microbial functions associated with methane metabolism after long-term 3-NOP supplementation in beef cattle. Fig. S11. Topological roles of rumen microbial taxa derived from co-occurrence networks after short-term and long-term 3-NOP supplementation. [file 40104_2025_1291_MOESM2_ESM.zip › 40104_2025_1291/Choi et al. R2 Additional file 2, Figure S3.pdf]

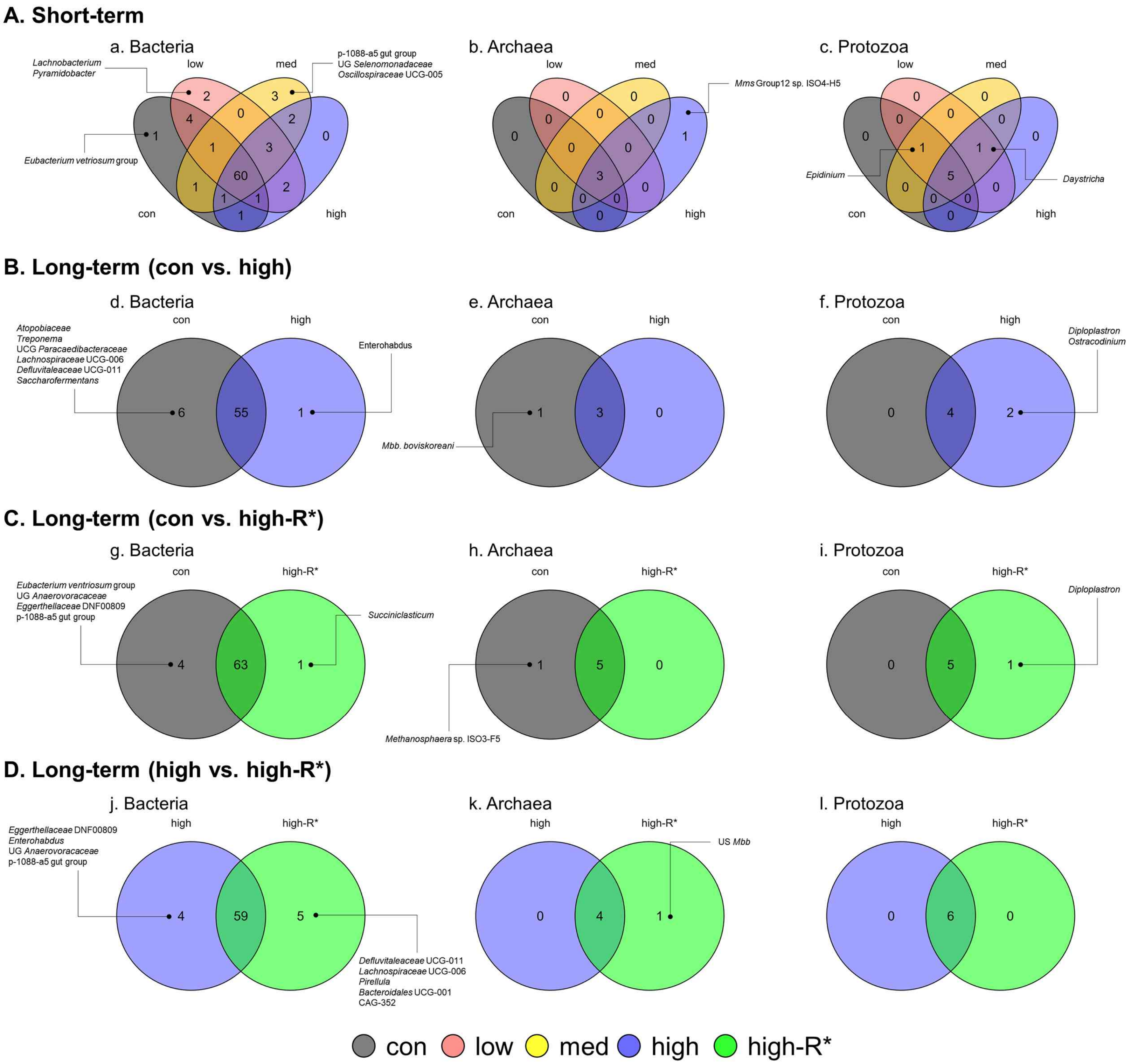
A. Short-term
a. Bacteria
low
med
con
high
Lachnobacterium
Pyramidobacter
p-1088-a5 gut group
UG Selenomonadaceae
Oscillospiraceae UCG-005
Eubacterium vetriosum group
2
3
4
0
2
1
1
3
0
60
1
2
1
1
1
b. Archaea
low
med
con
high
Mms Group12 sp. ISO4-H5
0
0
0
0
0
0
1
0
3
0
0
0
0
0
0
c. Protozoa
low
med
con
high
Epidinium
Daystricha
0
0
0
0
0
0
1
1
0
5
0
0
0
0
0
B. Long-term (con vs. high)
d. Bacteria
con
high
Atopobiaceae
Treponema
UCG Paracaedibacteraceae
Lachnospiraceae UCG-006
Defluvitaleaceae UCG-011
Saccharofermentans
Enterohabdus
6
55
1
e. Archaea
con
high
Mbb. boviskoreani
1
3
0
f. Protozoa
con
high
Diploplastron
Ostracodinium
0
4
2
C. Long-term (con vs. high-R*)
g. Bacteria
con
high-R*
Eubacterium ventriosum group
UG Anaerovoracaceae
Eggerthellaceae DNF00809
p-1088-a5 gut group
Succiniclasticum
4
63
1
h. Archaea
con
high-R*
Methanosphaera sp. ISO3-F5
1
5
0
i. Protozoa
con
high-R*
Diploplastron
0
5
1
D. Long-term (high vs. high-R*)
j. Bacteria
high
high-R*
Eggerthellaceae DNF00809
Enterohabdus
UG Anaerovoracaceae
p-1088-a5 gut group
Defluvitaleaceae UCG-011
Lachnospiraceae UCG-006
Pirellula
Bacteroidales UCG-001
CAG-352
4
59
5
k. Archaea
high
high-R*
US Mbb
0
4
1
l. Protozoa
high
high-R*
0
6
0
con
low
med
high
high-R*
