# Supplementary figures and images for: Short- and long-term dietary supplementation as well as withdrawal of the enteric methane inhibitor 3-nitrooxypropanol reveal distinct effects on the rumen microbial community
Source: J Anim Sci Biotechnol. 2025 Dec 1;16:162. doi: 10.1186/s40104-025-01291-w (PMC12667093; doi:10.1186/s40104-025-01291-w)

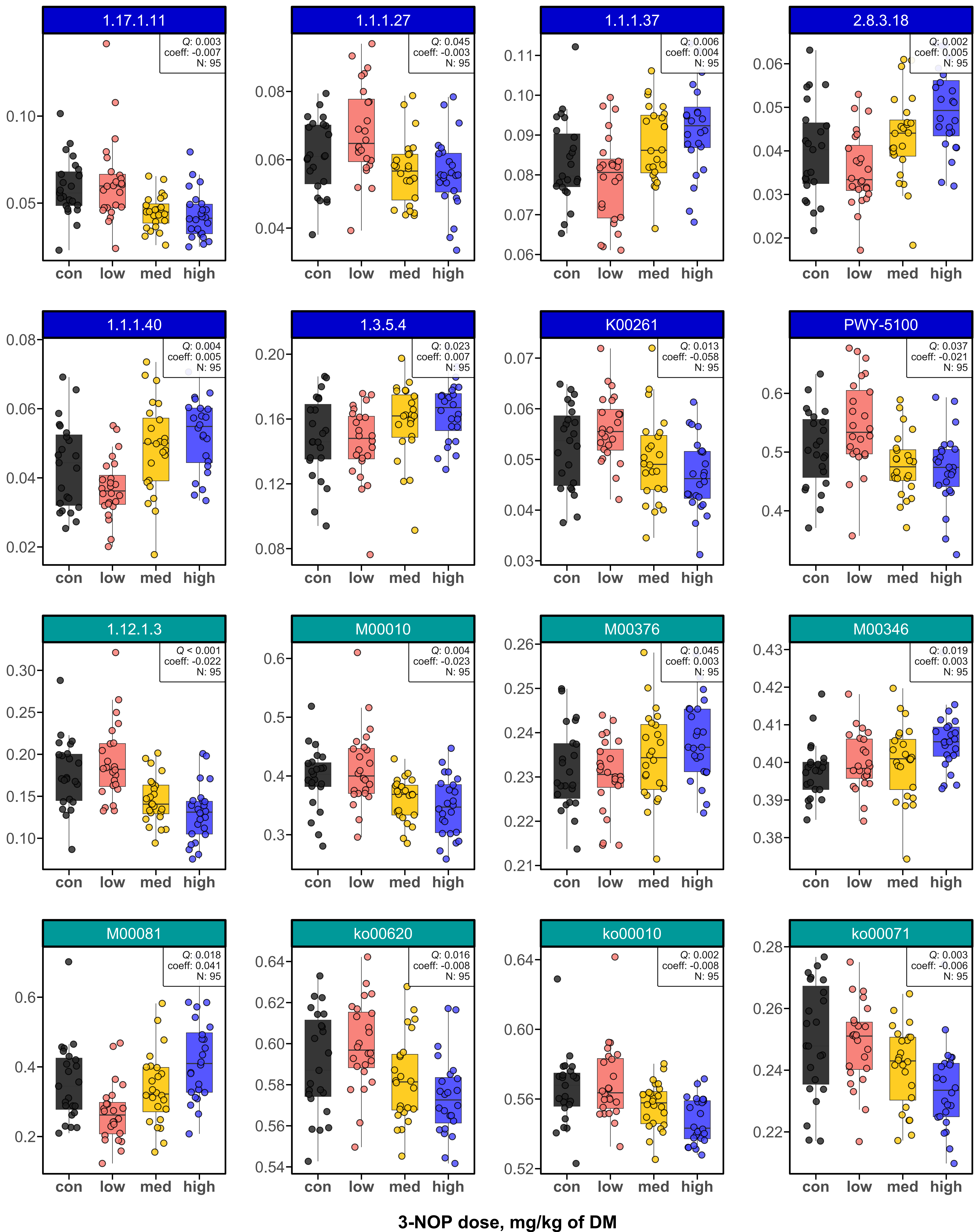

Supplement: Supplementary file 2 — Additional file 3: Fig. S1. Rarefaction curves of bacterial, archaeal, and protozoal communities after short- and long-term 3-NOP supplementation in beef cattle. Fig. S2. Comparison of alpha (Shannon index) and beta (Bray–Curtis dissimilarity) diversities of bacterial, archaeal, and protozoal communities in short-term (ST) and long-term (LT) periods. Fig. S3. Venn diagrams showing the genera of rumen microbes shared between and unique to short- and long-term studies. Fig. S4. Comparison of rumen microbial taxa after short-term 3-NOP supplementation in beef cattle. Fig. S5. Comparison of rumen microbial taxa between control and high groups after long-term 3-NOP supplementation in beef cattle. Fig. S6. Comparison of rumen microbial taxa between control and high-R* (recovery) groups after long-term 3-NOP supplementation in beef cattle. Fig. S7. Comparison of rumen microbial taxa between high and high-R* (recovery) groups after long-term 3-NOP supplementation in beef cattle. Fig. S8. Comparison of rumen microbial taxa after 3-NOP supplementation in beef cattle in short- and long-term periods. Fig. S9. Differentially abundant predicted microbial functions associated with methane metabolism after short-term 3-NOP supplementation in beef cattle. Fig. S10. Differentially abundant predicted microbial functions associated with methane metabolism after long-term 3-NOP supplementation in beef cattle. Fig. S11. Topological roles of rumen microbial taxa derived from co-occurrence networks after short-term and long-term 3-NOP supplementation. [file 40104_2025_1291_MOESM2_ESM.zip › 40104_2025_1291/Choi et al. Figure S7.pdf]

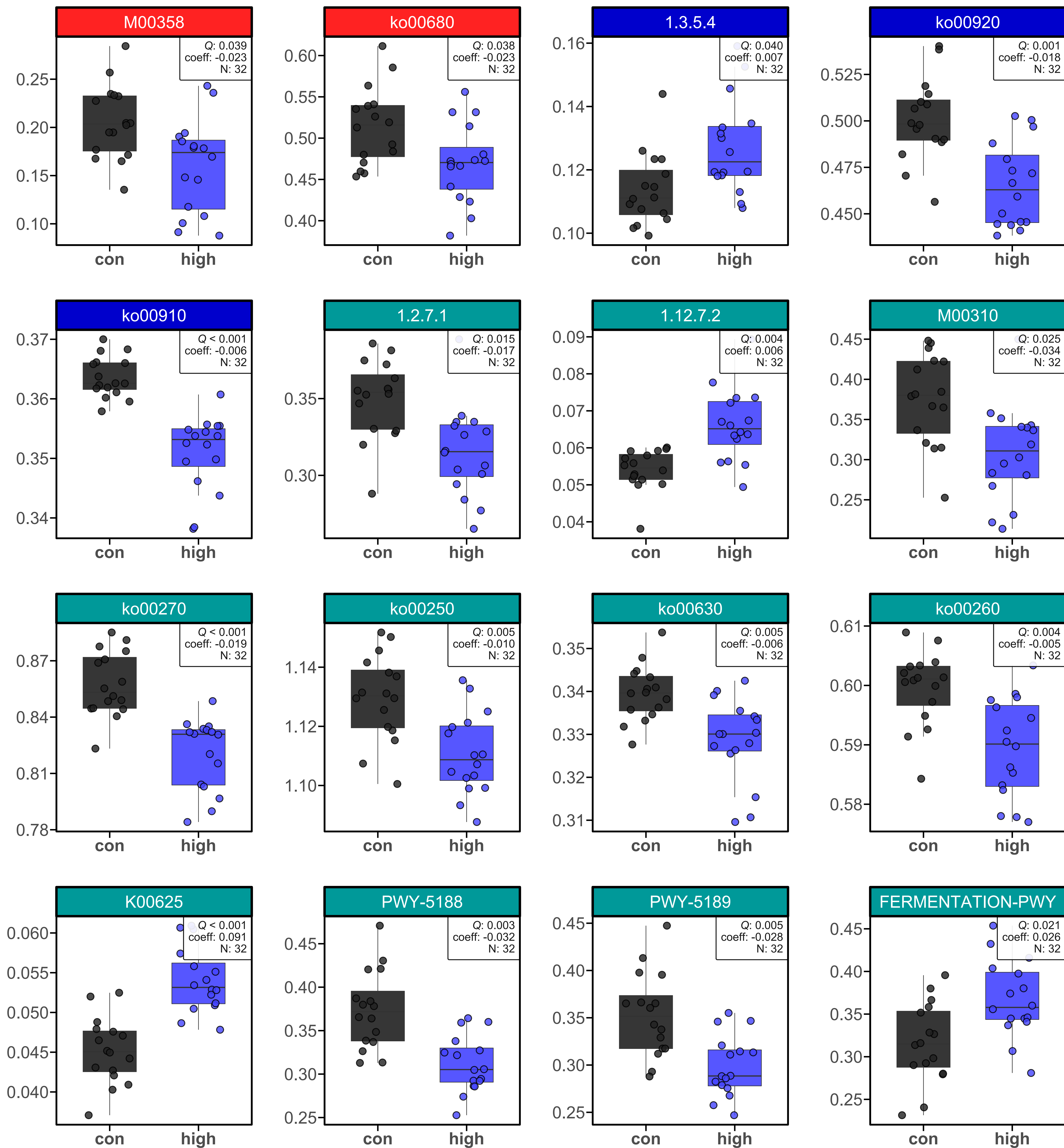

Supplement: Supplementary file 2 — Additional file 3: Fig. S1. Rarefaction curves of bacterial, archaeal, and protozoal communities after short- and long-term 3-NOP supplementation in beef cattle. Fig. S2. Comparison of alpha (Shannon index) and beta (Bray–Curtis dissimilarity) diversities of bacterial, archaeal, and protozoal communities in short-term (ST) and long-term (LT) periods. Fig. S3. Venn diagrams showing the genera of rumen microbes shared between and unique to short- and long-term studies. Fig. S4. Comparison of rumen microbial taxa after short-term 3-NOP supplementation in beef cattle. Fig. S5. Comparison of rumen microbial taxa between control and high groups after long-term 3-NOP supplementation in beef cattle. Fig. S6. Comparison of rumen microbial taxa between control and high-R* (recovery) groups after long-term 3-NOP supplementation in beef cattle. Fig. S7. Comparison of rumen microbial taxa between high and high-R* (recovery) groups after long-term 3-NOP supplementation in beef cattle. Fig. S8. Comparison of rumen microbial taxa after 3-NOP supplementation in beef cattle in short- and long-term periods. Fig. S9. Differentially abundant predicted microbial functions associated with methane metabolism after short-term 3-NOP supplementation in beef cattle. Fig. S10. Differentially abundant predicted microbial functions associated with methane metabolism after long-term 3-NOP supplementation in beef cattle. Fig. S11. Topological roles of rumen microbial taxa derived from co-occurrence networks after short-term and long-term 3-NOP supplementation. [file 40104_2025_1291_MOESM2_ESM.zip › 40104_2025_1291/Choi et al. R1 Figure S10.pdf]

**A**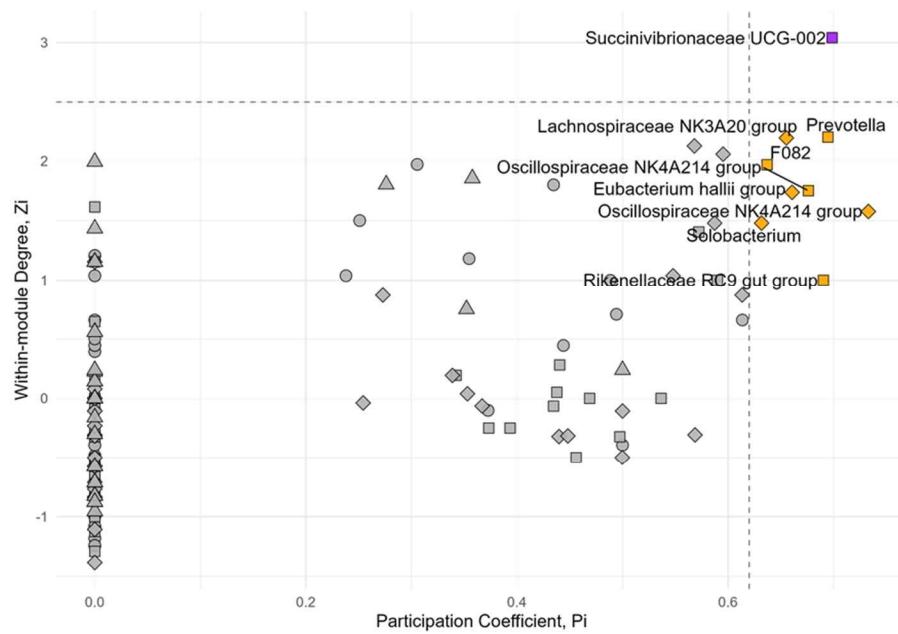**B**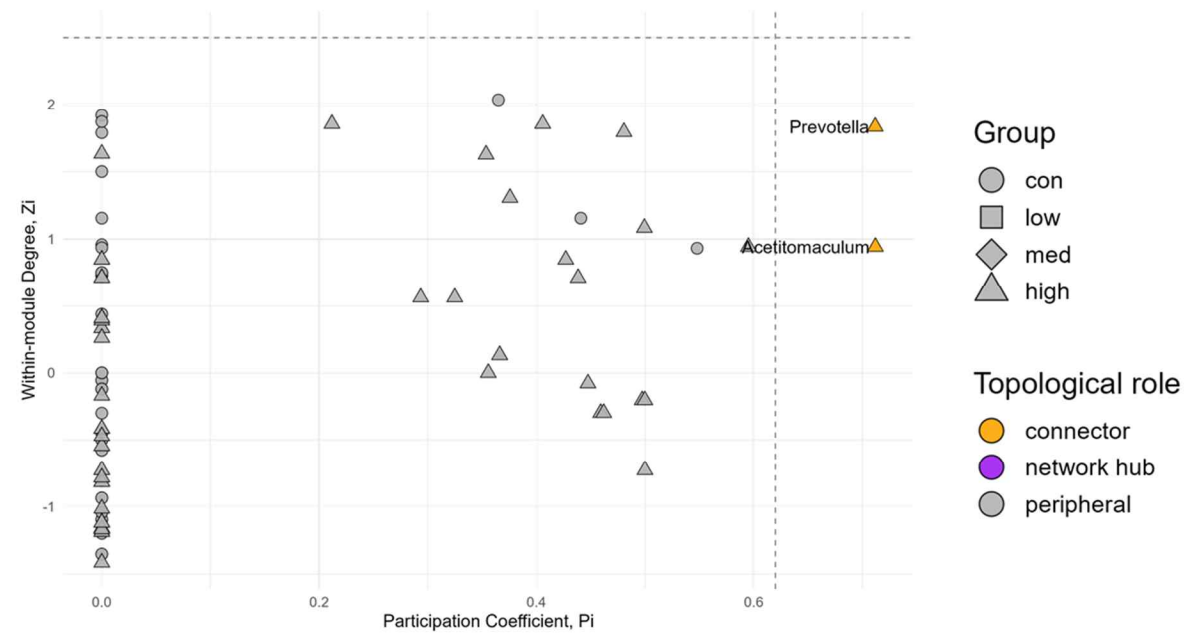

Supplement: Supplementary file 2 — Additional file 3: Fig. S1. Rarefaction curves of bacterial, archaeal, and protozoal communities after short- and long-term 3-NOP supplementation in beef cattle. Fig. S2. Comparison of alpha (Shannon index) and beta (Bray–Curtis dissimilarity) diversities of bacterial, archaeal, and protozoal communities in short-term (ST) and long-term (LT) periods. Fig. S3. Venn diagrams showing the genera of rumen microbes shared between and unique to short- and long-term studies. Fig. S4. Comparison of rumen microbial taxa after short-term 3-NOP supplementation in beef cattle. Fig. S5. Comparison of rumen microbial taxa between control and high groups after long-term 3-NOP supplementation in beef cattle. Fig. S6. Comparison of rumen microbial taxa between control and high-R* (recovery) groups after long-term 3-NOP supplementation in beef cattle. Fig. S7. Comparison of rumen microbial taxa between high and high-R* (recovery) groups after long-term 3-NOP supplementation in beef cattle. Fig. S8. Comparison of rumen microbial taxa after 3-NOP supplementation in beef cattle in short- and long-term periods. Fig. S9. Differentially abundant predicted microbial functions associated with methane metabolism after short-term 3-NOP supplementation in beef cattle. Fig. S10. Differentially abundant predicted microbial functions associated with methane metabolism after long-term 3-NOP supplementation in beef cattle. Fig. S11. Topological roles of rumen microbial taxa derived from co-occurrence networks after short-term and long-term 3-NOP supplementation. [file 40104_2025_1291_MOESM2_ESM.zip › 40104_2025_1291/Choi et al. R1 Figure S11.pdf]

A. Bacteria

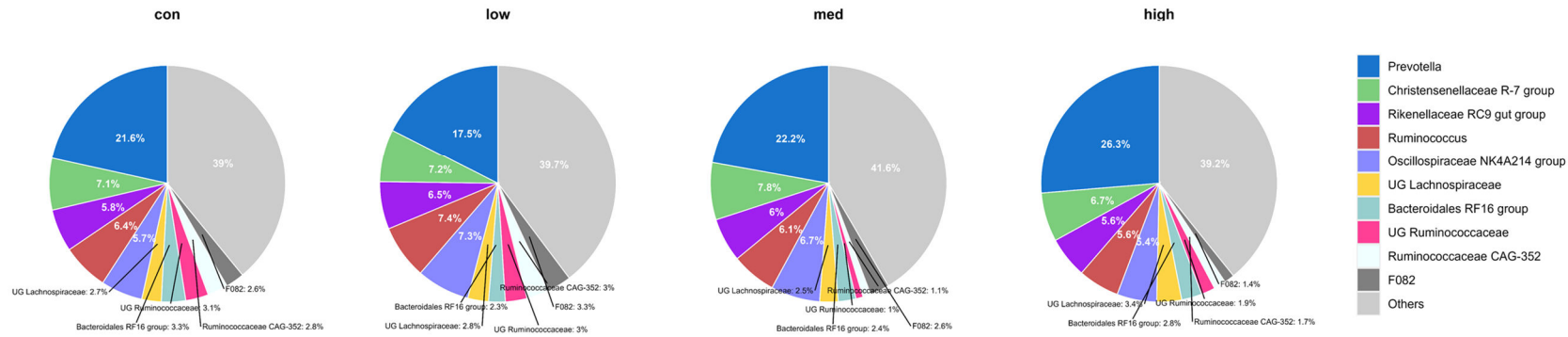

B. Archaea

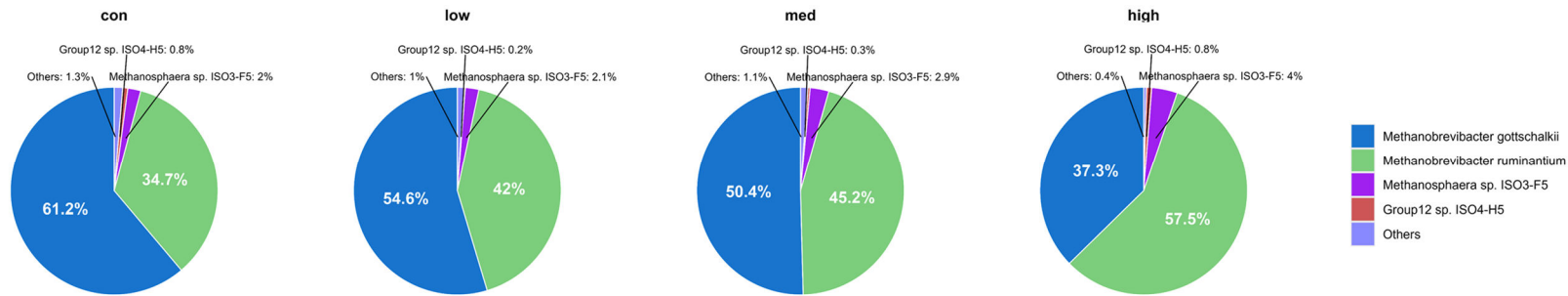

C. Protozoa

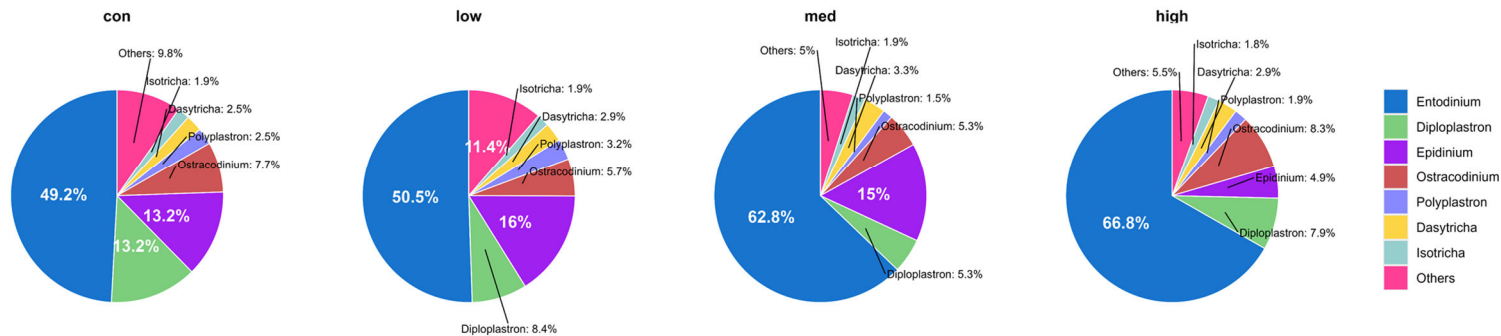

Supplement: Supplementary file 2 — Additional file 3: Fig. S1. Rarefaction curves of bacterial, archaeal, and protozoal communities after short- and long-term 3-NOP supplementation in beef cattle. Fig. S2. Comparison of alpha (Shannon index) and beta (Bray–Curtis dissimilarity) diversities of bacterial, archaeal, and protozoal communities in short-term (ST) and long-term (LT) periods. Fig. S3. Venn diagrams showing the genera of rumen microbes shared between and unique to short- and long-term studies. Fig. S4. Comparison of rumen microbial taxa after short-term 3-NOP supplementation in beef cattle. Fig. S5. Comparison of rumen microbial taxa between control and high groups after long-term 3-NOP supplementation in beef cattle. Fig. S6. Comparison of rumen microbial taxa between control and high-R* (recovery) groups after long-term 3-NOP supplementation in beef cattle. Fig. S7. Comparison of rumen microbial taxa between high and high-R* (recovery) groups after long-term 3-NOP supplementation in beef cattle. Fig. S8. Comparison of rumen microbial taxa after 3-NOP supplementation in beef cattle in short- and long-term periods. Fig. S9. Differentially abundant predicted microbial functions associated with methane metabolism after short-term 3-NOP supplementation in beef cattle. Fig. S10. Differentially abundant predicted microbial functions associated with methane metabolism after long-term 3-NOP supplementation in beef cattle. Fig. S11. Topological roles of rumen microbial taxa derived from co-occurrence networks after short-term and long-term 3-NOP supplementation. [file 40104_2025_1291_MOESM2_ESM.zip › 40104_2025_1291/Choi et al. R1 Figure S4.pdf]

## A. Bacteria

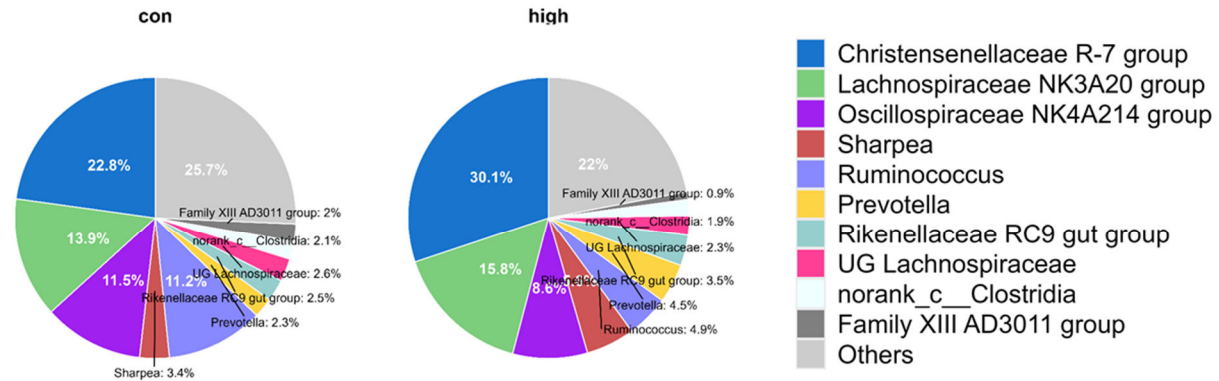

## B. Archaea

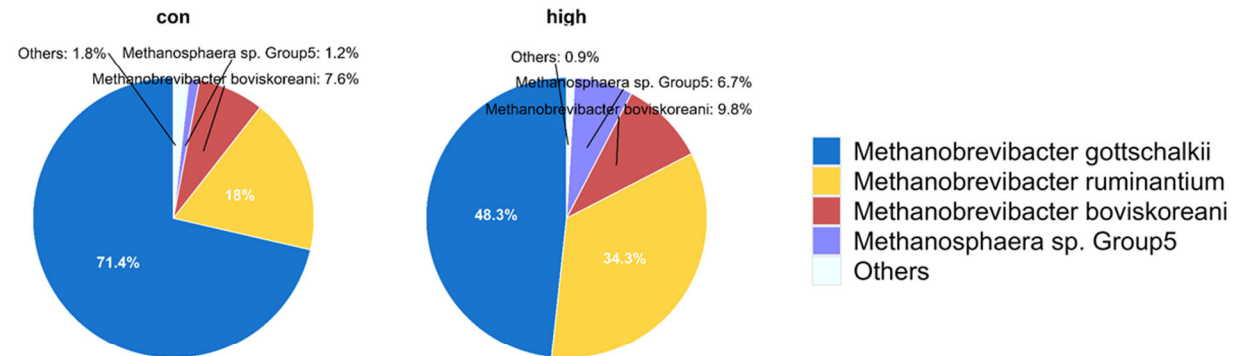

## C. Protozoa

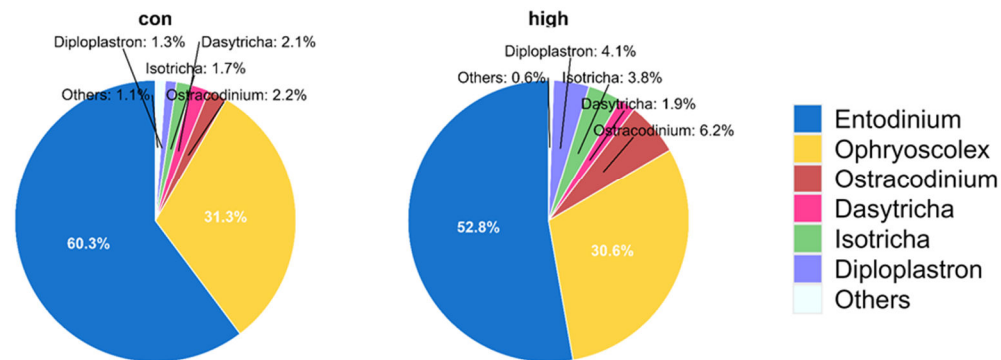

Supplement: Supplementary file 2 — Additional file 3: Fig. S1. Rarefaction curves of bacterial, archaeal, and protozoal communities after short- and long-term 3-NOP supplementation in beef cattle. Fig. S2. Comparison of alpha (Shannon index) and beta (Bray–Curtis dissimilarity) diversities of bacterial, archaeal, and protozoal communities in short-term (ST) and long-term (LT) periods. Fig. S3. Venn diagrams showing the genera of rumen microbes shared between and unique to short- and long-term studies. Fig. S4. Comparison of rumen microbial taxa after short-term 3-NOP supplementation in beef cattle. Fig. S5. Comparison of rumen microbial taxa between control and high groups after long-term 3-NOP supplementation in beef cattle. Fig. S6. Comparison of rumen microbial taxa between control and high-R* (recovery) groups after long-term 3-NOP supplementation in beef cattle. Fig. S7. Comparison of rumen microbial taxa between high and high-R* (recovery) groups after long-term 3-NOP supplementation in beef cattle. Fig. S8. Comparison of rumen microbial taxa after 3-NOP supplementation in beef cattle in short- and long-term periods. Fig. S9. Differentially abundant predicted microbial functions associated with methane metabolism after short-term 3-NOP supplementation in beef cattle. Fig. S10. Differentially abundant predicted microbial functions associated with methane metabolism after long-term 3-NOP supplementation in beef cattle. Fig. S11. Topological roles of rumen microbial taxa derived from co-occurrence networks after short-term and long-term 3-NOP supplementation. [file 40104_2025_1291_MOESM2_ESM.zip › 40104_2025_1291/Choi et al. R1 Figure S5.pdf]

## A. Bacteria

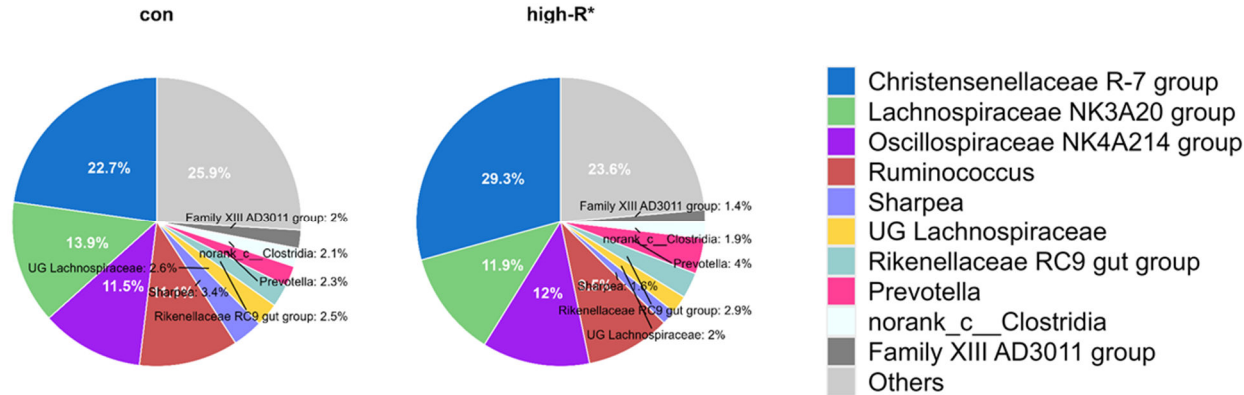

## B. Archaea

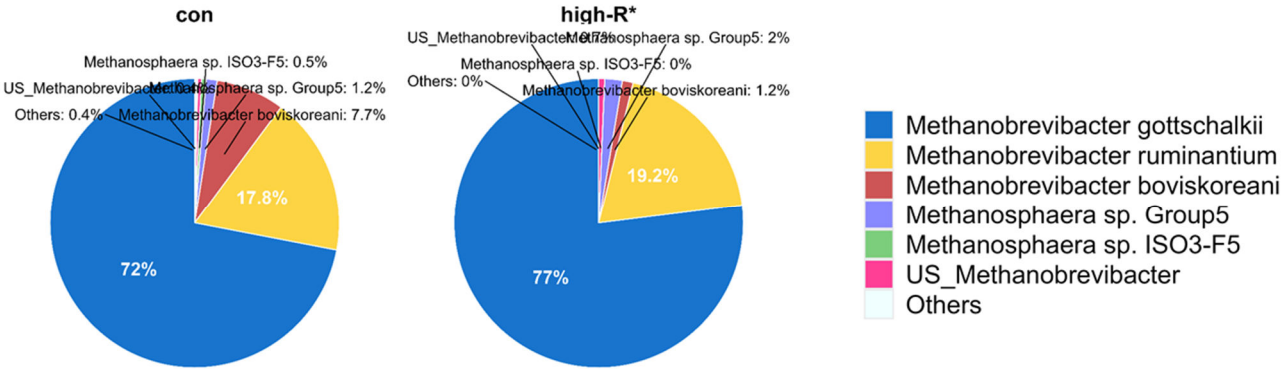

## C. Protozoa

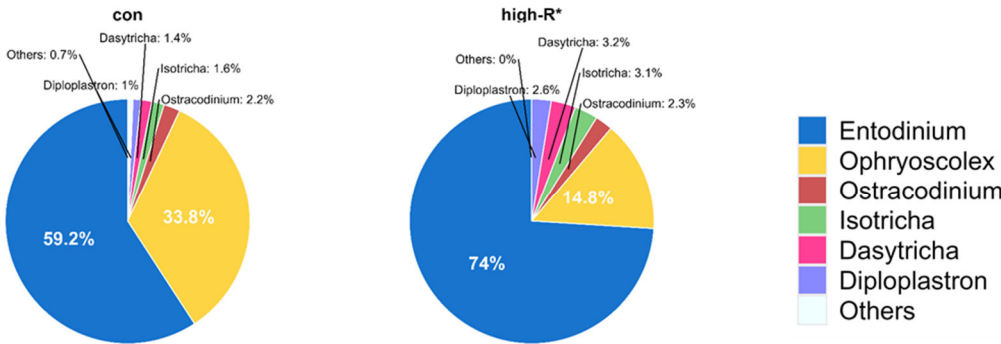

Supplement: Supplementary file 2 — Additional file 3: Fig. S1. Rarefaction curves of bacterial, archaeal, and protozoal communities after short- and long-term 3-NOP supplementation in beef cattle. Fig. S2. Comparison of alpha (Shannon index) and beta (Bray–Curtis dissimilarity) diversities of bacterial, archaeal, and protozoal communities in short-term (ST) and long-term (LT) periods. Fig. S3. Venn diagrams showing the genera of rumen microbes shared between and unique to short- and long-term studies. Fig. S4. Comparison of rumen microbial taxa after short-term 3-NOP supplementation in beef cattle. Fig. S5. Comparison of rumen microbial taxa between control and high groups after long-term 3-NOP supplementation in beef cattle. Fig. S6. Comparison of rumen microbial taxa between control and high-R* (recovery) groups after long-term 3-NOP supplementation in beef cattle. Fig. S7. Comparison of rumen microbial taxa between high and high-R* (recovery) groups after long-term 3-NOP supplementation in beef cattle. Fig. S8. Comparison of rumen microbial taxa after 3-NOP supplementation in beef cattle in short- and long-term periods. Fig. S9. Differentially abundant predicted microbial functions associated with methane metabolism after short-term 3-NOP supplementation in beef cattle. Fig. S10. Differentially abundant predicted microbial functions associated with methane metabolism after long-term 3-NOP supplementation in beef cattle. Fig. S11. Topological roles of rumen microbial taxa derived from co-occurrence networks after short-term and long-term 3-NOP supplementation. [file 40104_2025_1291_MOESM2_ESM.zip › 40104_2025_1291/Choi et al. R1 Figure S6.pdf]

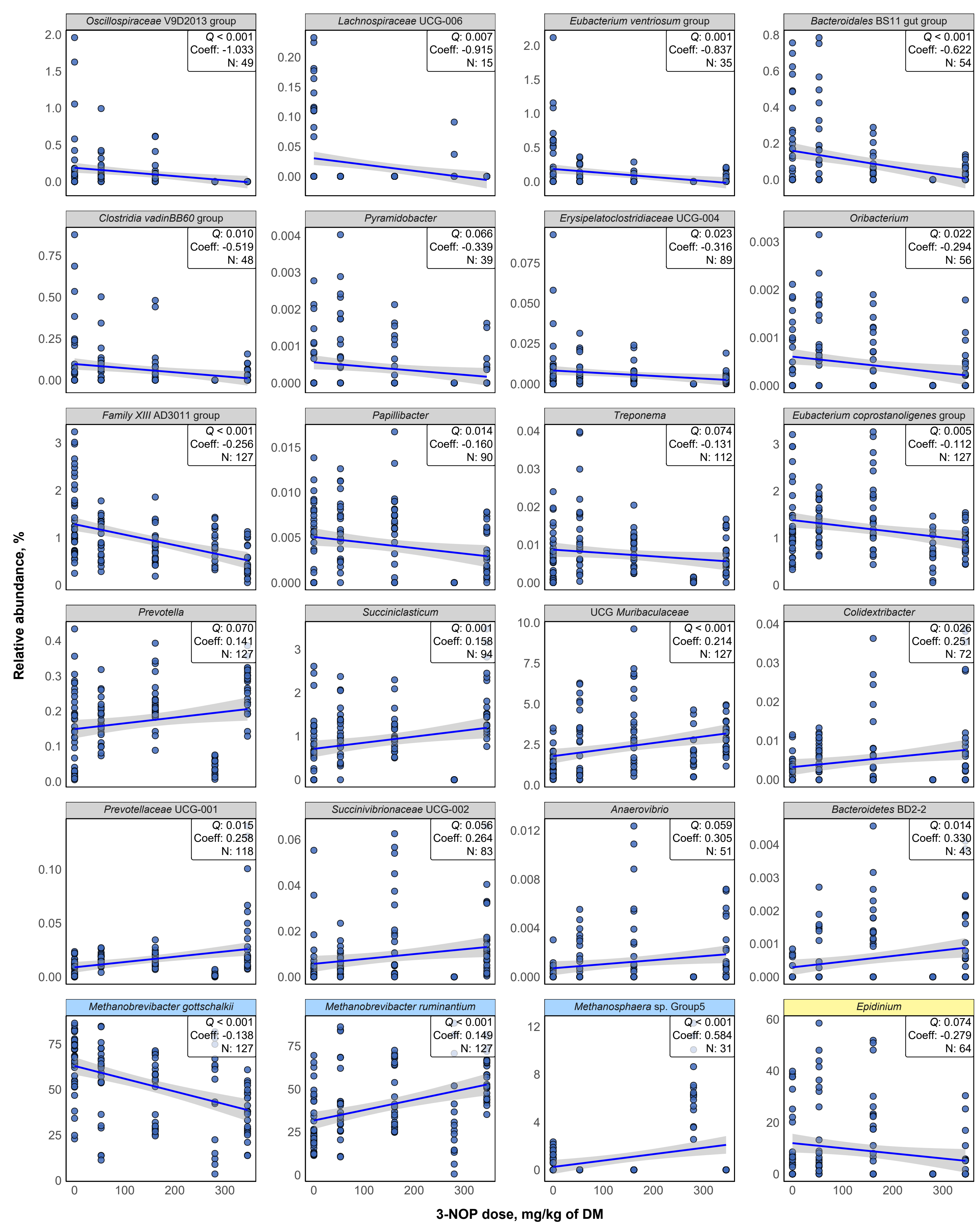

Supplement: Supplementary file 2 — Additional file 3: Fig. S1. Rarefaction curves of bacterial, archaeal, and protozoal communities after short- and long-term 3-NOP supplementation in beef cattle. Fig. S2. Comparison of alpha (Shannon index) and beta (Bray–Curtis dissimilarity) diversities of bacterial, archaeal, and protozoal communities in short-term (ST) and long-term (LT) periods. Fig. S3. Venn diagrams showing the genera of rumen microbes shared between and unique to short- and long-term studies. Fig. S4. Comparison of rumen microbial taxa after short-term 3-NOP supplementation in beef cattle. Fig. S5. Comparison of rumen microbial taxa between control and high groups after long-term 3-NOP supplementation in beef cattle. Fig. S6. Comparison of rumen microbial taxa between control and high-R* (recovery) groups after long-term 3-NOP supplementation in beef cattle. Fig. S7. Comparison of rumen microbial taxa between high and high-R* (recovery) groups after long-term 3-NOP supplementation in beef cattle. Fig. S8. Comparison of rumen microbial taxa after 3-NOP supplementation in beef cattle in short- and long-term periods. Fig. S9. Differentially abundant predicted microbial functions associated with methane metabolism after short-term 3-NOP supplementation in beef cattle. Fig. S10. Differentially abundant predicted microbial functions associated with methane metabolism after long-term 3-NOP supplementation in beef cattle. Fig. S11. Topological roles of rumen microbial taxa derived from co-occurrence networks after short-term and long-term 3-NOP supplementation. [file 40104_2025_1291_MOESM2_ESM.zip › 40104_2025_1291/Choi et al. R1 Figure S8.pdf]

## A. Short-term

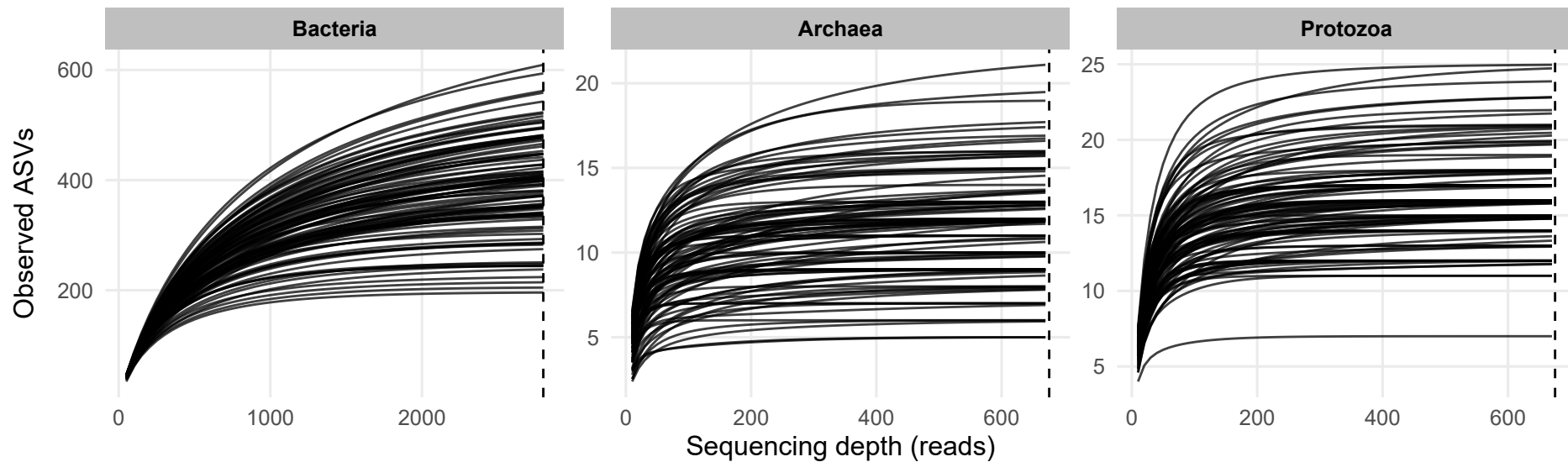

## B. Long-term

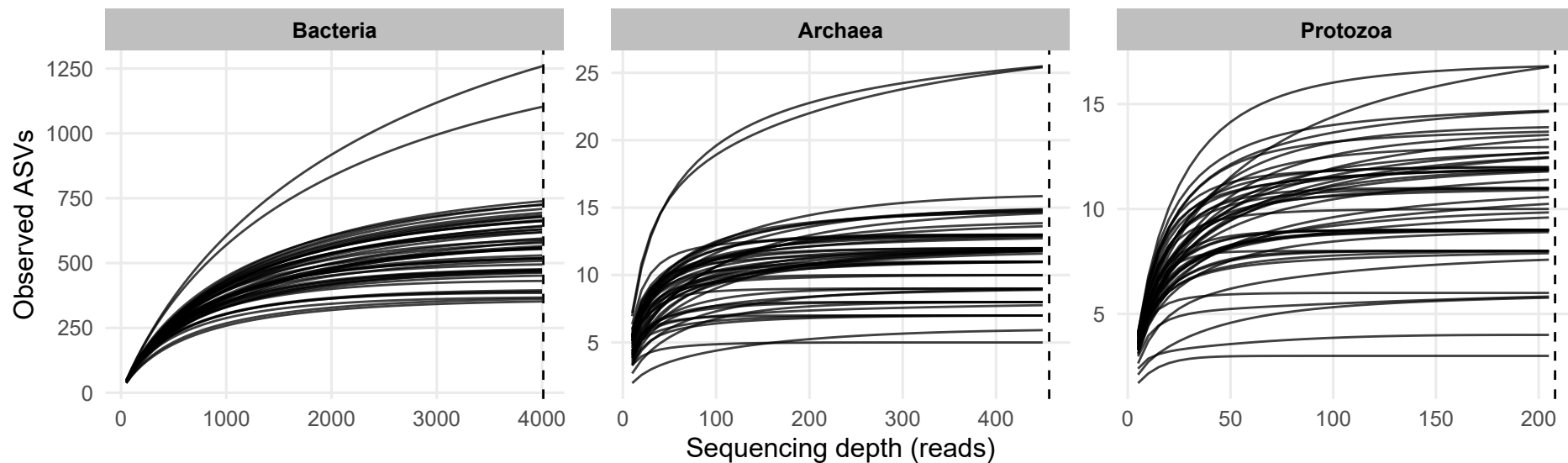

Supplement: Supplementary file 2 — Additional file 3: Fig. S1. Rarefaction curves of bacterial, archaeal, and protozoal communities after short- and long-term 3-NOP supplementation in beef cattle. Fig. S2. Comparison of alpha (Shannon index) and beta (Bray–Curtis dissimilarity) diversities of bacterial, archaeal, and protozoal communities in short-term (ST) and long-term (LT) periods. Fig. S3. Venn diagrams showing the genera of rumen microbes shared between and unique to short- and long-term studies. Fig. S4. Comparison of rumen microbial taxa after short-term 3-NOP supplementation in beef cattle. Fig. S5. Comparison of rumen microbial taxa between control and high groups after long-term 3-NOP supplementation in beef cattle. Fig. S6. Comparison of rumen microbial taxa between control and high-R* (recovery) groups after long-term 3-NOP supplementation in beef cattle. Fig. S7. Comparison of rumen microbial taxa between high and high-R* (recovery) groups after long-term 3-NOP supplementation in beef cattle. Fig. S8. Comparison of rumen microbial taxa after 3-NOP supplementation in beef cattle in short- and long-term periods. Fig. S9. Differentially abundant predicted microbial functions associated with methane metabolism after short-term 3-NOP supplementation in beef cattle. Fig. S10. Differentially abundant predicted microbial functions associated with methane metabolism after long-term 3-NOP supplementation in beef cattle. Fig. S11. Topological roles of rumen microbial taxa derived from co-occurrence networks after short-term and long-term 3-NOP supplementation. [file 40104_2025_1291_MOESM2_ESM.zip › 40104_2025_1291/Choi et al. R2 Additional file 2, Figure S1.pdf]
